# Multi-source Learning via Completion of Block-wise Overlapping Noisy Matrices

**Published:** 2023

**Authors:** Doudou Zhou, Tianxi Cai, Junwei Lu

**Affiliations:** Department of Biostatistics, Harvard T.H. Chan School of Public Health, Boston, Massachusetts 02115, USA; Department of Biostatistics, Harvard T.H. Chan School of Public Health, Boston, Massachusetts 02115, USA; Department of Biostatistics, Harvard T.H. Chan School of Public Health, Boston, Massachusetts 02115, USA

**Keywords:** Word embedding, data integration, singular value decomposition, transfer learning

## Abstract

Electronic healthcare records (EHR) provide a rich resource for healthcare research. An important problem for the efficient utilization of the EHR data is the representation of the EHR features, which include the unstructured clinical narratives and the structured codified data. Matrix factorization-based embeddings trained using the summary-level co-occurrence statistics of EHR data have provided a promising solution for feature representation while preserving patients’ privacy. However, such methods do not work well with multi-source data when these sources have overlapping but non-identical features. To accommodate multi-sources learning, we propose a novel word embedding generative model. To obtain multi-source embeddings, we design an efficient **B**lock-wise **O**verlapping **N**oisy **M**atrix **I**ntegration (BONMI) algorithm to aggregate the multi-source pointwise mutual information matrices optimally with a theoretical guarantee. Our algorithm can also be applied to other multi-source data integration problems with a similar data structure. A by-product of BONMI is the contribution to the field of matrix completion by considering the missing mechanism other than the entry-wise independent missing. We show that the entry-wise missing assumption, despite its prevalence in the works of matrix completion, is not necessary to guarantee recovery. We prove the statistical rate of our estimator, which is comparable to the rate under independent missingness. Simulation studies show that BONMI performs well under a variety of configurations. We further illustrate the utility of BONMI by integrating multi-lingual multi-source medical text and EHR data to perform two tasks: (i) co-training semantic embeddings for medical concepts in both English and Chinese and (ii) the translation between English and Chinese medical concepts. Our method shows an advantage over existing methods.

## 1. Introduction

### 1.1 Background

Electronic health records (EHR) have been playing a more and more important role in healthcare research ranging from disease phenotyping (Ahuja et al., 2020; De Freitas et al., 2021; Liu et al., 2021) to precision medicine (Raghu et al., 2017; Parbhoo et al., 2017; Sonabend et al., 2020; Zhou et al., 2022b). Despite the translational potential of EHR data, generating reliable real-world evidence from EHR data has been highly challenging, in part due to the significant heterogeneity across multiple healthcare centers. One approach to generalizability and reproducibility is through consensus learning with multiple EHR (multi-EHR). However, harmonizing data from multi-EHR for consensus learning is a major roadblock due to the lack of interoperability across healthcare systems (Rajkomar et al., 2018). The same clinical concepts can be represented by distinct codes or even different languages such as English and Chinese at different healthcare systems (Hernandez et al., 2009; Abhyankar et al., 2012). As a result, it is common for multiple data sources to have overlapping but non-identical clinical codes.

Although common data model has been increasingly adopted to improve interoperability across healthcare systems, significant discrepancies remain since most healthcare systems have adopted some but not all existing ontologies such as the International Classification of Disease (ICD) codes for diseases, Current Procedural Terminology (CPT) for procedures (Hirsch et al., 2015), and Logical Observation Identifiers Names and Codes (LOINC) for laboratory tests (Weeks and Pardee, 2019). One approach to harmonize data is to map local EHR codes to common ontologies (Kume et al., 2019; Tournavitis et al., 2009; Baloukas et al., 2010). Such an approach, generally requiring some level of manual effort and domain knowledge is thus time and resource-intensive and not scalable (Baorto et al., 1998; Kume et al., 2019). An alternative approach to data harmonization is through representation learning, which has been highly successful in natural language processing (Mikolov et al., 2013a). If a unified set of embeddings can be trained for all EHR codes across multiple systems, these embedding vectors can bridge data from different sources and hence achieve harmonization.

For training unified embeddings for all clinical concepts, the traditional one-hot approach will suffer from the curse of dimension. To train embeddings for a large number of concepts with observed relationship pairs, knowledge graph-based approaches (Wang et al., 2014; Yao et al., 2019) have been shown as highly effective. However, large-scale EHR data are typically represented as sequences of encounters over time. Based on co-occurrence patterns of EHR codes, neural network-based methods such as the skip-gram algorithm (Mikolov et al., 2013a; Pennington et al., 2014; Lin et al., 2019; Boag and Kané, 2017) have been proposed. Training of the skip-gram algorithm can also be equivalently achieved via performing singular value decomposition (SVD) on a pointwise mutual information (PMI) matrix derived from co-occurrence summaries of EHR data (Levy and Goldberg, 2014; Beam et al., 2019; Hong et al., 2021). The SVD-PMI algorithm is particularly appealing due to its scalability and privacy-preserving since it only requires simple summary-level EHR data, which can be shared across multiple systems.

However, these existing methods are ineffective in co-training embeddings for multi-EHR data when the EHR codes from multiple sources overlap but are not identical. Neural network methods that require patient-level data are not feasible due to data-sharing constraints. The simple pre-training approach effectively assumes that code pairs that do not appear in the same health system have low similarity, which is a poor assumption since two distinct codes can represent the same clinical concepts in two systems due to coding heterogeneity. To better accommodate coding heterogeneity, one may separately train embeddings within each EHR source and then follow up with an alignment step such that codes shared at multiple systems have similar embeddings after alignment (Smith et al., 2017; Kementchedjhieva et al., 2018; Conneau et al., 2018). However, such two-step methods are not efficient both computationally and statistically and are less generalizable to settings with more than two sources.

In this paper, we propose the **B**lock-wise **O**verlapping **N**oisy **M**atrix **I**ntegration (BONMI) algorithm to co-train embeddings for clinical concepts from multiple sources. The BONMI algorithm is built on top of a novel generative model similar to Arora et al. (2016, 2018) but allows EHR codes or concepts to belong to multiple sources. Specifically, let $w_{t}^{\left(s,i\right)}$ denote the code the *i*th patient from the *s*th source receives at time *t*. We assume that the probability of the code $w_{t}^{\left(s,i\right)}$ taking value *w* is $P(w_{t}^{\left(s,i\right)}=w|c_{t}^{\left(s,i\right)})\propto exp(x_{w}^{⊤}c_{t}^{\left(s,i\right)})$, where ${\text{x}}_{w}$ is the underlying embedding representation of the specific code *w* assumed to be the same for all sources *w* occurs, and ${\text{c}}_{t}^{\left(s,i\right)}$ is some latent vector with a random walk on the sphere associated with each patient in each source. Our model allows the multiple sources to have overlapping but not identical corpora.

Under the proposed generative model, the SVD-PMI estimator consistently estimates the underlying embeddings when there is a single data source (Arora et al., 2018; Lu et al., 2023). With multiple sources, the PMI of code pairs that do not co-occur in the same system is not observable. Under the proposed generative model, the underlying embeddings for each code do not depend on the source and can be recovered based on data from the source that contains the code. However, it is no longer feasible to estimate the embeddings through simple decomposition of PMI matrices due to the missingness of co-pairs that do not co-occur. We propose an efficient estimation procedure based on the low-rank nature of the PMI matrices and the low-dimensional embedding vectors. Our idea connects to the orthogonal Procrustes problem (Gower and Dijksterhuis, 2004; Schönemann, 1966; Gower, 1975), which has been widely used to align embeddings across languages in the machine translation (Kementchedjhieva et al., 2018; Smith et al., 2017; Conneau et al., 2018; Søgaard et al., 2018; Xing et al., 2015). We use an orthogonal transformation to align the eigenspace of the two sub-matrices through their overlap, then complete the missing entries by the inner products of the two low-rank components. Moreover, we generalize our method to the multiple sources scenario by applying the method to each pair of the sub-matrices. Since BONMI operates on matrices from any two sources, it is suitable for parallel computing.

### 1.2 Related Literature

Related works to BONMI can be classified into two categories: (i) matrix completion and (ii) multi-source data integration. Matrix completion aims to recover a low-rank matrix given a subset of its entries which may be corrupted by noise (Keshavan et al., 2010; Candès and Recht, 2009). It has received considerable attention due to the diverse applications such as collaborative filtering (Hu et al., 2008; Rennie and Srebro, 2005) and recommendation systems. As reviewed in Nguyen et al. (2019), diverse algorithms have been proposed including Frobenius norm minimization (Lee and Bresler, 2010), alternative minimization (Haldar and Hernando, 2009; Tanner and Wei, 2016; Wen et al., 2012), optimization over smooth Riemannian manifold (Vandereycken, 2013) and stochastic gradient descent (Koren et al., 2009; Takács et al., 2007; Paterek, 2007; Sun and Luo, 2016; Ge et al., 2016, 2017; Du et al., 2017; Ma et al., 2018). However, most existing literature on matrix completion assumed that the observed entries are independently sampled (Keshavan et al., 2010; Chen and Wainwright, 2015; Candes and Plan, 2010; Candès and Tao, 2010; Mazumder et al., 2010; Chen, 2015; Keshavan et al., 2010; Chen and Wainwright, 2015; Zheng and Lafferty, 2016; Fazel, 2002; Candès and Recht, 2009; Cai et al., 2010; Tanner and Wei, 2013; Combettes and Pesquet, 2011; Jain et al., 2010, e.g.), which does not hold in the current setting as the missingness will always be block-wise.

On the other hand, many works on multi-source data integration analysis needed to deal with the block-wise missingness for downstream analyses, such as model selection (Xue and Qu, 2020), principal component analysis (PCA) (Cai et al., 2016; Zhu et al., 2018), classification (Yuan et al., 2012; Xiang et al., 2014) and prediction (Yu et al., 2020). However, these methods do not apply to the current problem since they need to use the patient-level data and have additional model assumptions such as a classification model (Yuan et al., 2012; Xiang et al., 2014) or a regression model (Yu et al., 2020; Xue and Qu, 2020). For example, Xue and Qu (2020) focused on the model selection when the covariates were block-wise missing due to incomplete observations. They assumed a linear model between the response and the covariates and showed the consistency of the estimation of the linear coefficients. Although our problem can also be modeled through a regression framework by using the observed entries to predict the missing entries, the independent assumption required by Xue and Qu (2020) will be violated. Specifically, Xue and Qu (2020) assumed not only that the covariates are independently sampled but also that the observation errors are independent and normally distributed. If we fit Xue and Qu (2020) to the current setting, both assumptions would be violated since the “covariates” and the errors are not independently sampled. Cai et al. (2016) proposed a structured matrix completion (SMC) algorithm that leverages the approximate low-rank structure to recover the missing off-diagonal sub-matrix efficiently. However, the SMC algorithm considers a noiseless scenario and does not allow for a multi-block missingness structure, ubiquitous in the integrative analysis of multi-source or multi-view data. Since SMC operates on a 2 × 2 block matrix with a missing block in the off-diagonal sub-matrices, it cannot fully utilize the observed information when applied to our problem. Approximation errors can also make SMC fail to perform well with a lack of theoretical guarantee. As demonstrated by our numerical studies, SMC performs poorly compared to BONMI in the presence of noise in the case of two sources.

### 1.3 Our Contribution

Our paper extends the word vector generative model in Arora et al. (2016) to accommodate multi-sources learning, which allows these sources to have overlapping but not identical entities. We design an efficient algorithm BONMI to aggregate the multi-source PMI matrices optimally with a theoretical guarantee to obtain multi-source embeddings. BONMI can also be applied to other multi-source data integration problems with a similar data structure. A by-product of BONMI is the contribution to the field of matrix completion by considering the missing mechanism other than the entry-wise independent missing. We show that the entry-wise missing assumption, despite its prevalence in the works of matrix completion, is not necessary to guarantee recovery. We prove the statistical rate of our estimator, which is comparable to the rate under the independently missing assumption (Ma et al., 2018; Chen and Wainwright, 2015; Negahban and Wainwright, 2012; Koltchinskii et al., 2011).

The rest of the paper is organized as follows. In Section 2, we introduce in detail the proposed BONMI method. The theoretical properties of BONMI are analyzed in Section 3. Simulation results are shown in Section 4 to investigate the numerical performance of the proposed method. A real data application is given in Section 5. Section 6 extends the model to asymmetric matrices and concludes the paper. For space reasons, the proofs of the main results are given in the supplement. In addition, some key technical tools used in the proof of the main theorems are also developed and proved in the supplement.

## 2. Methodology

### 2.1 Notations

We first introduce some notations. We use bold-faced symbols to represent vectors and matrices. For any vector ***v***, ‖v‖ denotes its Euclidean norm. For any matrix $A\in R^{d\times q}$, we let $\sigma_{j}(A)$ and $\lambda_{j}(A)$ (if *d* = *q*) denote its respective *j*th largest singular value and eigenvalue. The smallest singular value $\sigma_{min(m,n)}(A)$ will be denoted by $\sigma_{min}(A)$. We let ‖A‖, $\|A{\|}_{F}$, $\|A{\|}_{2,\infty }$ and $\|A{\|}_{\infty }$ respectively denote the spectral norm (i.e., the largest singular value), the Frobenius norm, the $\ell_{2}/\ell_{\infty }$ norm (i.e., the largest $\ell_{2}$ norm of the rows), and the entry-wise $\ell_{\infty }$ norm (the largest magnitude of all entries) of **A**. We let $A_{j,}$ and $A_{.,j}$ denote the *j*th row and *j*th column of **A**, and let A(i,j) denote the (i,j) entry of **A**. Besides, we use the symbol ≡ to denote ‘defined to be.’ For any integer d≥1, we let [d]≡{1,…,d}. For indices sets $\Omega_{1}\subseteq [d]$ and $\Omega_{2}\subseteq [q]$, we use $A_{\Omega_{1},\Omega_{2}}$ to represent its sub-matrix with row indices $\Omega_{1}$ and column indices $\Omega_{2}$.

We let ${𝒪}^{n\times r}$ represent the set of all n×r orthonormal matrices. For a sub-Gaussian random variable *Y* , its sub-Gaussian norm is defined as $\left\|Y{\|}_{\psi_{2}}=inf\{t>0:Ee^{-Y^{2}/t^{2}}\leq 2\right\}$. We use the standard notation f(n)=O(g(n)) or f(n)≲g(n) to represent |f(n)|≤c|g(n)| for some constant c>0.

### 2.2 Model

We extend the log-linear model word production model proposed by Arora et al. (2016) to multiple sources. Assume that we have *m* sources, and in the *s*th source, we have $n_{s}$ independent sequences, which may be referred to as $n_{s}$ patients for EHR data. Let ${𝒱}_{s}$ be the word set of the *s*th source with size $N_{s}=\left|{𝒱}_{s}\right|$. For simplicity, we assume that each sequence has length *T*. For the *i*th sequence from the *s*th source, the code sequence is $\left\{w_{1}^{\left(s,i\right)},\ldots ,w_{T}^{\left(s,i\right)}\right\}$. In short, for each sequence in the *s*th source, the occurrence probability of a code *w* at time *t* is determined by its latent vector $x_{w}\in R^{r}$ and a discourse vector $c_{t}^{\left(s,i\right)}\in R^{r}$ with the random walk on the sphere. Specifically,

$$P(w_{t}^{\left(s,i\right)}=w|c_{t}^{\left(s,i\right)})=\frac{exp(x_{w}^{⊤}c_{t}^{\left(s,i\right)})}{\sum_{w^{\prime }\in {𝒱}_{s}}exp(x_{w^{\prime }}^{⊤}c_{t}^{\left(s,i\right)})}.$$

Under this generative model, we are interested in estimating the clinical codes’ embeddings ${\left\{x_{w}\right\}}_{w\in 𝒱*}$ based on the summary statistics only such as the co-occurrence matrices to preserve the privacy where $𝒱*=\cup_{s=1}^{m}{𝒱}_{s}$ is the corpus of the clinical features from the *m* sources. We further denote the corpus for all clinical features as 𝒱, where ${𝒱}_{s}\subset 𝒱$ is generated by a binomial model. For the *s*th source, the corpus ${𝒱}_{s}$ is a random subset of 𝒱 sampled as

(1) $$P\left(w\in {𝒱}_{s}\right)=p_{s}\in (0,1),\;\text{for}\;w\in 𝒱\;\text{and}\;s\in [m]\;\text{independently}.$$

Notice that it is not necessary to have 𝒱*=𝒱. This model for ${\left\{{𝒱}_{s}\right\}}_{s=1}^{m}$ allows the emergence of new features which may have not been included by the current sources, such as COVID-19 (Zhou et al., 2022a). When a new source is incorporated, some new features in $𝒱\backslash {𝒱}^{*}$ can occur in the new source. The PMI matrix $\mathrm{PMI}={\left\{PMI\left(w,w^{\prime }\right)\right\}}_{w,w^{\prime }\in 𝒱}$ of the population corpus 𝒱 is defined as

$$PMI\left(w,w^{\prime }\right)=log\frac{p\left(w,w^{\prime }\right)}{p(w)p\left(w^{\prime }\right)},\;\text{for}\;w,w^{\prime }\in 𝒱,$$

where p(w) is the occurrence probability of the word *w* and $p\left(w,w^{\prime }\right)$ is the co-occurrence probability of the words *w* and $w^{\prime }$. Arora et al. (2016) showed that $log\;p\left(w,w^{\prime }\right)=\|x_{w}+x_{w^{\prime }}{\|}^{2}/(2r)-2log\;Z+o(1)$ and $log\;p(w)={\left\|x_{w}\right\|}^{2}/(2r)-log\;Z+o(1)$ for some constant *Z*, then derived that $PMI\left(w,w^{\prime }\right)\approx x_{w}^{⊤}x_{w^{\prime }}/r$ (see, e.g., Theorem 2.2 of Arora et al. (2016) and Proposition 4.4 of Lu et al. (2023)), which implies the rationale of recovering word embeddings from the PMI matrices. With a bit of abuse of notation, we also define $x_{w}=x_{w}/\sqrt{r}$ as the word embedding. With a single hospital, **PMI**, while not directly observable, can be estimated empirically, and hence performing an SVD of the empirical **PMI** can lead to consistent estimators for $x_{w}$. However, in the settings where different hospitals have overlapping but non-identical codes, entries of **PMI** can not be directly estimated for code pairs that do not belong to the same hospital.

On the other hand, the principal sub-matrices of **PMI** can be estimated from each source. Let $p_{s}\left(w,w^{\prime }\right)$ be the co-occurrence probability of codes *w* and $w^{\prime }$ in windows of size *q* in the *s*th source, $p_{s}(w)=\sum_{w^{\prime }\in {𝒱}_{s}}p_{s}\left(w,w^{\prime }\right)$ and the population PMI matrix for the *s*th source ${PMI}_{s}\in R^{N_{s}\times N_{s}}$ as

$${PMI}_{s}\left(w,w^{\prime }\right)=log\frac{p_{s}\left(w,w^{\prime }\right)}{p_{s}(w)p_{s}\left(w^{\prime }\right)}\;\text{for}\;w,w^{\prime }\in {𝒱}_{s}.$$

The estimates of the PMI matrices using the co-occurrence statistics are

$${\hat{PMI}}_{s}\left(w,w^{\prime }\right)=log\frac{{𝒞}_{s}\left(w,w^{\prime }\right)}{{𝒞}_{s}(w,\cdot ){𝒞}_{s}\left(w^{\prime },\cdot \right)},\;\text{for}\;s\in [m],$$

where ${𝒞}_{s}=[{𝒞}_{s}{\left(w,w^{\prime }\right]}_{w,w^{\prime }\in {𝒱}_{s}}$ is the observed co-occurrence of word *w* with word $w^{\prime }$ in the window size *q* across all sequences of the *s*th source defined similar to Beam et al. (2019); Lu et al. (2023):

$${𝒞}_{s}\left(w,w^{\prime }\right)=|\{(t,h):|t-h|\leq q\;\text{and}\;w_{t}^{\left(s,i\right)}=w,w_{h}^{\left(s,i\right)}=w^{\prime }|t,h\in [T],i\in \left[n_{s}\right]\}|$$

and ${𝒞}_{s}(w,\cdot )=\sum_{w^{\prime }\in {𝒱}_{s}}{𝒞}_{s}\left(w,w^{\prime }\right)$. We then define the shifted positive PMI (SPPMI) matrix estimator as

$${\hat{SPMI}}_{s}\left(w,w^{\prime }\right)=max\{{\hat{PMI}}_{s}\left(w,w^{\prime }\right),\eta \},\;\text{for}\;s\in [m],$$

where η>−∞ is a given threshold value. In the theoretical analysis, one may show that ${PMI}_{s}\left(w,w^{\prime }\right)$ is lower bounded by some constant with high probability under appropriate assumptions (Lu et al., 2023). So the shifted positive PMI would be closer to the truth than the original PMI with a high probability if η is chosen properly. According to Levy and Goldberg (2014), the shifted positive PMI (SPPMI) can perform better than the PMI matrix, with the reasoning being that humans tend to more easily associate positive values (e.g. ‘Canada’ and ‘snow’) rather than negative ones (‘Canada’ and ‘desert’). The default choice for η is set as 0, meaning no shift and setting negative PMI values as 0. Empirically, we find that η=0 works well. For ease of presentation, we will use the positive PMI matrix (PPMI) matrix estimator as

$${\hat{PPMI}}_{s}\left(w,w^{\prime }\right)=max\{{\hat{PMI}}_{s}\left(w,w^{\prime }\right),0\},\;\text{for}\;s\in [m]$$

throughout the paper, while our theorems still hold for other choices of η. Let $X_{s}\in R^{N_{s}\times r}$ be the matrix whose rows are composed of the word embeddings in the *s*th source. Define the error matrix $E^{s}=\hat{PPMI}-X_{s}X_{s}^{⊤}$. The estimated PPMI matrices approximate the population PMI matrices thus approximating $X_{s}X_{s}^{⊤}$ such that

$${\left\|E^{s}\right\|}_{\infty }≲n_{s}^{-\frac{1}{4}}T^{-\frac{1}{2}}\;\text{and}\;\left\|E^{s}\right\|≲N_{s}n_{s}^{-\frac{1}{4}}T^{-\frac{1}{2}},$$

which follows straightforwardly from Lu et al. (2023) under some mild assumptions. In reality, $n_{s}$ can be sufficiently larger than $N_{s}$. For example, in the EHR system, the number of clinical codes is smaller than 10, 000 while the number of patients can be 23 million (Zhou et al., 2022a).

Without loss of generality, we assume that 𝒱=[N] and $𝒱*=\left[N_{0}\right]$, where $N_{0}=|𝒱*|$, otherwise we can rearrange the orders of codes. Let $X={\left(x_{1},\ldots ,x_{N}\right)}^{⊤}\in R^{N\times r}$ be the population embedding matrix and $W^{*}={XX}^{⊤}$. We then have

(2) $$W^{s}\equiv \hat{PPM}I_{s}=W_{s}^{*}+E^{s}={\left[W^{*}(i,j)\right]}_{j\in {𝒱}_{s}}^{i\in {𝒱}_{s}}+E^{s},\;\text{for}\;s\in [m]$$

by the definition of $E^{s}$. Define $\sigma_{s}=\left\|E^{s}\right\|/\sqrt{N_{s}}$. In our theoretical analysis, we only use the operator norm of the error matrices $E^{s}$. Therefore, our results can be applied to general matrix integration problems.

Our task is to first recover $W_{0}^{*}=W_{𝒱*,𝒱*}^{*}={\left[W^{*}(i,j)\right]}_{j\in 𝒱*}^{i\in 𝒱*}\in R^{N_{0}\times N_{0}}$, and then obtain the embeddings $X^{*}={\left(x_{1},\ldots ,x_{N_{0}}\right)}^{⊤}$ by performing SVD on the estimate of $W_{0}^{*}$. Let the eigendecomposition of $W^{*}$ be

(3) $$W^{*}=U^{*}\Sigma^{*}{\left(U^{*}\right)}^{⊤},$$

where $U^{*}\in R^{N\times r}$ consists of orthonormal columns, and $\Sigma^{*}$ is an r×r diagonal matrix with eigenvalues in a descending order, i.e., $\lambda_{max}=\lambda_{1}\geq \cdots \geq \lambda_{r}=\lambda_{min}>0$.

We first state two assumptions that are standard in existing literature (Candès and Recht, 2009; Ma et al., 2018) for sample complexity and the incoherence condition which basically assumes information is distributed uniformly among entries.

**Assumption 1 (Incoherence condition)** *The coherence coefficient of* $U^{*}$ *satisfies* $\mu_{0}=O(1)$, *where* $\mu_{0}=\mu (U^{*})=\frac{N}{r}{max}_{i\in [N]}\sum_{j=1}^{r}U^{*}(i,j{)}^{2}$.

**Assumption 2 (Sample complexity)** *The sampling probability* $p_{0}={min}_{s\in [m]}p_{s}$ *satisfies* $p_{0}\geq C\sqrt{\mu_{0}r\;logN/N}$ *for some sufficiently large constant* *C*. *Besides*, ${max}_{s\in [m]}p_{s}/p_{0}=O(1)$.

**Remark 1** *Based on the observed data, we can estimate* $W_{0}^{*}$ *but not* $W^{*}$, *as we have no information on* ${\left\{x_{w}\right\}}_{w\in 𝒱\backslash 𝒱*}$. *The inclusion of* $W^{*}$ *and the sampling* model (1) *of* ${\left\{{𝒱}_{s}\right\}}_{s=1}^{m}$ *serves as a convenient random setup and links to the matrix completion literature. In reality, the overlapping matrices are determined by dictionaries linking multiple sources or ontologies such as the ICD and CPT codes commonly adopted in the EHR. Under the sampling* model (1), $W_{0}^{*}$ *is a random sub-matrix of* $W^{*}$. *Instead of making assumptions on* $W_{0}^{*}$, *which is a random object, we impose assumptions on* $W^{*}$ *(i.e., Assumption 1 above and Assumption 4 in* Section 3).

### 2.3 An Ideal Case

To illustrate the BONMI algorithm, we first consider an ideal case that the error matrices ${\left\{E^{s}\right\}}_{s=1}^{m}$ are zero and we observe the truth ${\left\{W_{s}^{*}\right\}}_{s=1}^{m}$ when m=2. To simplify the notations, we denote $s\backslash k\equiv {𝒱}_{s}\backslash {𝒱}_{k}$ and $s\cap k\equiv {𝒱}_{s}\cap {𝒱}_{k}$ when they are used as the subscripts of a matrix, and recall that $W_{s}^{*}\equiv W_{{𝒱}_{s},{𝒱}_{s}}^{*}$. Assume the two sampled sub-matrices are $W_{s}^{*}$ and $W_{k}^{*}$. Since the singular values are invariant under row/column permutations, without loss of generality, we can rearrange our data matrices such that

(4) $$W_{s}^{*}=\left[\begin{array}{cc} W_{s\backslash k,s\backslash k}^{*} & W_{s\backslash k,s\cap k}^{*}\\ W_{s\cap k,s\backslash k}^{*} & W_{s\cap k,s\cap k}^{*} \end{array}\right];\;W_{k}^{*}=\left[\begin{array}{ll} W_{s\cap k,s\cap k}^{*} & W_{s\cap k,k\backslash s}^{*}\\ W_{k\backslash s,s\cap k}^{*} & W_{k\backslash s,k\backslash s}^{*} \end{array}\right]$$

and

(5) $$W_{0}^{*}=\left[\begin{array}{ccc} W_{s\backslash k,s\backslash k}^{*} & W_{s\backslash k,s\cap k}^{*} & W_{s\backslash k,k\backslash s}^{*}\\ W_{s\cap k,s\backslash k}^{*} & W_{s\cap k,s\cap k}^{*} & W_{s\cap k,k\backslash s}^{*}\\ W_{k\backslash s,s\backslash k}^{*} & W_{k\backslash s,s\cap k}^{*} & W_{k\backslash s,k\backslash s}^{*} \end{array}\right].$$

Recall that our goal is to recover $W_{0}^{*}$ based on the observed $W_{s}^{*}$ and $W_{k}^{*}$. This can be achieved by estimating the missing blocks $W_{s\backslash k,k\backslash s}^{*}$ and $W_{k\backslash s,s\backslash k}^{*}=W_{s\backslash k,k\backslash s}^{*⊤}$ by the symmetry of $W_{0}^{*}$. As the missing entries are block-wise, a theoretical guarantee based on the assumption of independent missing will fail in the current case. Instead, we propose a method based on the orthogonal transformation, which exploits the following proposition.

**Proposition 2** *Suppose* $W^{*}$ *has eigendecomposition* (3) *and satisfies Assumptions 1 and 2. Since* $max\{rank(W_{s}^{*}),rank(W_{k}^{*})\}\leq rank(W^{*})=r$, *we suppose the eigendecompositions of* $W_{s}^{*}$ *and* $W_{k}^{*}$ *are*

$$W_{s}^{*}=V_{s}^{*}\Sigma_{s}^{*}{\left(V_{s}^{*}\right)}^{⊤}\;\text{and}\;W_{k}^{*}=V_{k}^{*}\Sigma_{k}^{*}{\left(V_{k}^{*}\right)}^{⊤},$$

*where* $V_{s}^{*}$ *and* $V_{k}^{*}$ *are the eigenvectors of* $W_{s}^{*}$ *and* $W_{k}^{*}$, *respectively. We further decompose* $V_{s}^{*}$ *and* $V_{k}^{*}$ *as* $V_{s}^{*}={\left({\left(V_{s1}^{*}\right)}^{⊤},{\left(V_{s2}^{*}\right)}^{⊤}\right)}^{⊤}$, $V_{k}^{*}={\left({\left(V_{k1}^{*}\right)}^{⊤},{\left(V_{k2}^{*}\right)}^{⊤}\right)}^{⊤}$ *with* $V_{s2}^{*},V_{k1}^{*}\in R^{\left|{𝒱}_{s}\cap {𝒱}_{k}\right|\times r}$. *Then with probability at least* $1-O\left(1/N^{3}\right),W_{s\backslash k,k\backslash s}^{*}$ *in* (5) *can be exactly given by*

(6) $$W_{s\backslash k,k\backslash s}^{*}=V_{s1}^{*}{\left(\Sigma_{s}^{*}\right)}^{1/2}G({\left(\Sigma_{s}^{*}\right)}^{1/2}{\left(V_{s2}^{*}\right)}^{⊤}V_{k1}^{*}{\left(\Sigma_{k}^{*}\right)}^{1/2}){\left(\Sigma_{k}^{*}\right)}^{1/2}{\left(V_{k2}^{*}\right)}^{⊤},$$

*where* G(·) *is a matrix value function defined as:* $G(C)=HZ^{⊤}$ *with* $H\Omega Z^{⊤}$ *the SVD of* $C\in R^{r\times r}$.

Proposition 2 shows that, when there is no error, $W_{s\backslash k,k\backslash s}^{*}$ can be recovered precisely based on $W_{s}^{*}$ and $W_{k}^{*}$ with high probability. The proposition can be easily extended to the case when m>2.

### 2.4 BONMI Algorithm for Two Sources

When noise exists, we use the above idea but add an additional step of weighted average. Since it is possible to observe the entries of $W^{*}$ more than once due to multiple sources, the weighted average is a natural idea to reduce the variance of estimation in the existence of noise. In reality, heterogeneity always exists which means the errors of different sources may vary. As a result, we decide to use the weights inversely proportional to the error levels. We start with the case m=2 again. Currently, we decompose two overlapping matrices $W^{s}=W_{s}^{*}+E^{s}$ and $W^{k}=W_{k}^{*}+E^{k}$ as follows

$$W^{s}=\left[\begin{array}{cc} W_{s\backslash k,s\backslash k}^{s} & W_{s\backslash k,s\cap k}^{s}\\ W_{s\cap k,s\backslash k}^{s} & W_{s\cap k,s\cap k}^{s} \end{array}\right],\;W^{k}=\left[\begin{array}{cc} W_{s\cap k,s\cap k}^{k} & W_{s\cap k,k\backslash s}^{k}\\ W_{k\backslash s,s\cap k}^{k} & W_{k\backslash s,k\backslash s}^{k} \end{array}\right],\;\text{for}\;1\leq s<k\leq m.$$

Then we can combine $W_{s}$ and $W_{k}$ to obtain

(7) $$\tilde{W}=\left[\begin{array}{ccc} W_{s\backslash k,s\backslash k}^{s} & W_{s\backslash k,s\cap k}^{s} & 0\\ W_{s\cap k,s\backslash k}^{s} & W_{s\cap k,s\cap k}^{a} & W_{s\cap k,k\backslash s}^{k}\\ 0 & W_{k\backslash s,s\cap k}^{k} & W_{k\backslash s,k\backslash s}^{k} \end{array}\right],$$

where $W_{s\cap k,s\cap k}^{a}\equiv \alpha_{s}W_{s\cap k,s\cap k}^{s}+\alpha_{k}W_{s\cap k,s\cap k}^{k}$ is the weighted average of the overlapping part with $\alpha_{i}>0$, i=s,k and $\alpha_{s}+\alpha_{k}=1$. The weights should ideally depend on the strength of the error matrices, $E^{s}$ and $E^{k}$, to optimize estimation. We detail the estimation of the weights in Section 2.5. To estimate $W_{s\backslash k,k\backslash s}^{*}$, let

$${\tilde{W}}_{s}=\left[\begin{array}{cc} W_{s\backslash k,s\backslash k}^{s} & W_{s\backslash k,s\cap k}^{s}\\ W_{s\cap k,s\backslash k}^{s} & W_{s\cap k,s\cap k}^{a} \end{array}\right]\;\text{and}\;{\tilde{W}}_{k}=\left[\begin{array}{cc} W_{s\cap k,s\cap k}^{a} & W_{s\cap k,k\backslash s}^{k}\\ W_{k\backslash s,s\cap k}^{k} & W_{k\backslash s,k\backslash s}^{k} \end{array}\right],$$

and the rank-*r* eigendecompositions of ${\tilde{W}}_{s}$ and ${\tilde{W}}_{k}$ be ${\tilde{V}}_{s}{\tilde{\Sigma }}_{s}{\tilde{V}}_{s}^{⊤}$ and ${\tilde{V}}_{k}{\tilde{\Sigma }}_{k}{\tilde{V}}_{k}^{⊤}$, respectively. Specifically, ${\tilde{V}}_{s}$ and ${\tilde{V}}_{k}$ can be decomposed block-wise such that ${\tilde{V}}_{s}={\left({\tilde{V}}_{s1}^{⊤},{\tilde{V}}_{s2}^{⊤}\right)}^{⊤}$ and ${\tilde{V}}_{k}={\left({\tilde{V}}_{k1}^{⊤},{\tilde{V}}_{k2}^{⊤}\right)}^{⊤}$ where ${\tilde{V}}_{s2},{\tilde{V}}_{k1}\in R^{\left|{𝒱}_{s}\cap {𝒱}_{k}\right|\times r}$. So the estimator of $W_{s\backslash k,k\backslash s}^{*}$ is

(8) $${\tilde{W}}_{sk}={\tilde{V}}_{s1}{\tilde{\Sigma }}_{s}^{1/2}G\left({\tilde{\Sigma }}_{s}^{1/2}{\tilde{V}}_{s2}^{⊤}{\tilde{V}}_{k1}{\tilde{\Sigma }}_{k}^{1/2}\right){\tilde{\Sigma }}_{k}^{1/2}{\tilde{V}}_{k2}^{⊤},$$

according to the Proposition 2. After getting ${\tilde{W}}_{sk}$, we impute it back to $\tilde{W}$ to obtain

(9) $$\hat{W}=\left[\begin{array}{ccc} W_{s\backslash k,s\backslash k}^{s} & W_{s\backslash k,s\cap k}^{s} & {\tilde{W}}_{sk}\\ W_{s\cap k,s\backslash k}^{s} & W_{s\cap k,s\cap k}^{a} & W_{s\cap k,k\backslash s}^{k}\\ {\tilde{W}}_{sk}^{⊤} & W_{k\backslash s,s\cap k}^{k} & W_{k\backslash s,k\backslash s}^{k} \end{array}\right].$$

Then we can obtain the rank-*r* eigendecomposition of $\hat{W}$, denoted as ${\hat{W}}_{r}=\hat{U}\hat{\Sigma }{\hat{U}}^{⊤}$, as an estimate of $W_{0}^{*}$.

### 2.5 BONMI Algorithm

We next introduce the BONMI algorithm for recovering $W_{0}^{*}$, based on m≥2 PPMI matrices ${\left\{W^{s}\right\}}_{s\in [m]}$. Our algorithm consists of three main steps: (I) aggregation of the *m* matrices, (II) estimation of missing parts, and (III) low-rank approximation, as summarized in Algorithm 1.


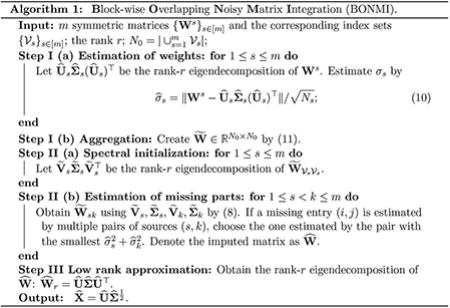


#### Step I: Aggregation.

We first aggregate ${\left\{W^{s}\right\}}_{s\in [m]}$ to obtain $\tilde{W}$ similar to the m=2 case, which requires an estimation for the weights ${\left\{\alpha_{s}\right\}}_{s\in [m]}$. Similar to standard meta-analysis, the optimal weight for the *s*th source can be chosen as $\sigma_{s}^{-2}$. We estimate $\sigma_{s}$ as by ${\hat{\sigma }}_{s}=\left\|W^{s}-{\hat{U}}_{s}{\hat{\Sigma }}_{s}{\hat{U}}_{s}^{⊤}\right\|/\sqrt{N_{s}}$, where ${\hat{U}}_{s}{\hat{\Sigma }}_{s}{\hat{U}}_{s}^{⊤}$ is the rank-*r* eigendecomposition of $W^{s}$. We then create the matrix $\tilde{W}\in R^{n\times n}$ as follows

(11) $$\tilde{W}\left(i,j\right)=\sum_{s=1}^{m}\alpha_{ij}^{s}W^{s}(v_{i}^{s},v_{j}^{s})𝟙\left(i,j\in {𝒱}_{s}\right),$$

for all pairs of (i,j) such that ${𝒮}_{ij}\equiv \sum_{s=1}^{m}𝟙\left(i,j\in {𝒱}_{s}\right)>0$, where $v_{i}^{s}$ denotes the row(column) index in $W^{s}$ corresponding to the *i*th row (column) of $W_{0}^{*}$, and $\alpha_{ij}^{s}=\frac{1}{{\overset{ˆ}{\sigma }}_{s}^{2}}(\sum_{k=1}^{m}𝟙(i,j\in {{𝒱}_{k}){\overset{ˆ}{\sigma }}_{k}^{-2})}^{-1}$. The entries in the missing blocks with ${𝒮}_{ij}=0$ are initialized as zero.

**Remark 3** *It is natural to use the inverse of noise variances as the weights to aggregate multiple observations, for instance, weighted least squares (*Ruppert and Wand, 1994*). Here we follow the same routine, and the choice is direct since, intuitively, in this way, we can minimize the variance of the noise of the overlapping matrices. A formal analysis is provided in* Section S.2.3.

#### Step II: Imputation.

We next impute the missing entries with ${𝒮}_{ij}=0$. For 1≤s≤k≤m, we impute the entries of $\tilde{W}$ corresponding to $\left({𝒱}_{s}\backslash {𝒱}_{k}\right)\times \left({𝒱}_{k}\backslash {𝒱}_{s}\right)$ using ${\tilde{W}}_{s}\equiv {\tilde{W}}_{{𝒱}_{s},{𝒱}_{s}}$ and ${\tilde{W}}_{k}$ the same way as (8). If a missing entry (i,j) can be estimated by multiple pairs of sources (s,k), we choose the one estimated by the pair with the smallest ${\hat{\sigma }}_{s}^{2}+{\hat{\sigma }}_{k}^{2}$. After Steps I and II, all missing entries of $\tilde{W}$ are imputed, and we denote the imputed matrix as $\hat{W}$.

**Remark 4** *When a missing entry can be estimated from multiple source pairs, it may be desirable to use a weighted average of these estimates. However, determining an optimal set of weights is more challenging in this case compared to Step I because the variances of these estimates are difficult to estimate. We instead choose the estimate based on the source pairs with the highest overall quality as measured by* ${\hat{\sigma }}_{s}^{2}+{\hat{\sigma }}_{k}^{2}$.

#### Step III: Low-raxnk approximation.

Finally, we factorize $\hat{W}$ by rank-*r* eigendecomposition to obtain the final estimator: ${\hat{W}}_{r}\equiv \hat{U}\hat{\Sigma }{\hat{U}}^{⊤}$.

**Remark 5 (Computational complexity)** *The main computational cost of the BONMI algorithm comes from the eigendecomposition, which is of order* $O(\sum_{s=1}^{m}{\left|{𝒱}_{s}\right|}^{2}r)$. *The estimation step involves matrix multiplication and a singular value decomposition of a* r×r *matrix for each source pair* *s and k*, *resulting in a computational cost of order* $O\left(\sum_{1\leq s<k\leq m}\left|{𝒱}_{s}\right|\left|{𝒱}_{k}\right|r\right)$. *Thus, the overall computational cost is of order*

$$O\left(\sum_{s=1}^{m}{\left|{𝒱}_{s}\right|}^{2}r+\sum_{1\leq s<k\leq m}\left|{𝒱}_{s}\right|\left|{𝒱}_{k}\right|r\right)=O\left({\left(\sum_{s=1}^{m}\left|{𝒱}_{s}\right|\right)}^{2}r\right).$$

*In comparison, the computational complexity of the gradient descent-based algorithms is* $O\left(T{\left(\sum_{s=1}^{m}\left|{𝒱}_{s}\right|\right)}^{2}r\right)$*, where T is the iteration complexity dependent on the pre-set precision* ϵ*. Existing algorithms set* T=n/rlog(1/ϵ) (Sun and Luo, 2016), $T=r^{2}log(1/\epsilon )$ (Chen and Wainwright, 2015*), and* T=log(1/ϵ) (Ma et al., 2018*). Thus, the BONMI algorithm is more computationally efficient compared to these algorithms*.

## 3. Theoretical Analysis

In this section, we investigate the theoretical properties of the algorithm. We first present some general assumptions required by our theorems. To this end, we define the condition number $\tau \equiv \lambda_{1}(W^{*})/\lambda_{r}(W^{*})=\lambda_{max}/\lambda_{min}$. Besides, we need conditions to bound the noise strength and the condition number.

**Assumption 3** *Let* $\sigma \equiv {max}_{s\in [m]}\sigma_{s}$. *Then* σ *satisfies* $\sigma \ll \lambda_{min}\sqrt{p_{0}/N}$.

**Assumption 4** $\tau \equiv \lambda_{1}(W^{*})/\lambda_{r}(W^{*})=\lambda_{max}/\lambda_{min}=O(1)$. *Throughout this paper, we assume the condition number is bounded by a fixed constant, independent of the problem size (i.e., N and r).*

**Remark 6** *Assumptions 1.4 are standard assumptions in many existing literature* (Ma et al., 2018; Chen and Wainwright, 2015; Negahban and Wainwright, 2012; Koltchinskii et al., 2011), *whereas different rates are required. Specifically, in Assumption 2, we only require the sampling probability to be of the order* $O(\sqrt{logN/N})$, *which can tend to zero when the population size tends to infinity. In our setting, the sample size of each source is about* $N^{2}p_{0}^{2}$. *Then we have* $N^{2}p_{0}^{2}\geq C^{2}\mu_{0}rN\;log\;N$. *Relatively*, Ma et al. (2018) *requires that the sample size satisfies* $N^{2}p\geq C\mu_{0}^{3}r^{3}N\;{log}^{3}\;N$ *for some sufficiently large constant* C>0 *where p is the entrywise sampling probability. In Assumption 3, the sampling probability* $p_{0}$ *and the eigenvalue* $\lambda_{min}$ *can vary with N. Compared to* Ma et al. (2018), *they require the noise satisfies* $\sigma \ll \lambda_{min}\sqrt{\frac{p}{N\kappa^{3}\mu_{0}r\;{log}^{3}N}}$. *Our signal-to-noise ratio assumption has the same order as theirs up to some constants and log factors since* $\mu_{0}$, *r, and* κ *are assumed to be constants*.

The parameter of interest is $W_{0}^{*}$ with eigendecomposition $W_{0}^{*}=X^{*}{\left(X^{*}\right)}^{⊤}=U_{0}^{*}\Sigma_{0}^{*}{\left(U_{0}^{*}\right)}^{⊤}$. Let $\hat{X}=\hat{U}{\hat{\Sigma }}^{1/2}$ be the output of Algorithm 1 and define $K=r\mu_{0}\tau$. The upper bound for the estimation errors of $X^{*}$ (which is identifiable up to an orthogonal transformation) and hence $W_{0}^{*}$ under the special case of m=2 is presented in Theorem 7.

**Theorem 7** *Under Assumptions 1, 2, 3, and 4, when* m=2, *with probability at least* $1-O\left(N^{-3}\right)$, *there exists* $O_{X}\in {𝒪}^{r\times r}$ *such that*

- *if* $p_{0}=o(1/logN)$ *or* $p_{0}$ *is bounded away from* 0, *we have*
(12) $$\left\|\hat{X}O_{X}-X^{*}\right\|≲\frac{\left\{\left(1-p_{0}\right)K^{2}+1\right\}K}{\sqrt{\lambda_{min}}}\sqrt{N}\sigma ;$$
- otherwise,
(13) $$\left\|\hat{X}O_{X}-X^{*}\right\|≲\frac{\left\{\left(1-p_{0}\right)K^{2}\left(p_{0}logN\right)+1\right\}K}{\sqrt{\lambda_{min}}}\sqrt{N}\sigma .$$

**Remark 8** *Besides the spectral norm upper bound for* $\hat{X}$*, we can also obtain upper bound of other metrics such as* $\left\|\hat{X}{\hat{X}}^{⊤}-X^{*}{\left(X^{*}\right)}^{⊤}\right\|$, ${\left\|\hat{X}O_{X}-X^{*}\right\|}_{F}$ *and* ${\left\|\hat{X}{\hat{X}}^{⊤}-X^{*}{\left(X^{*}\right)}^{⊤}\right\|}_{F}$ *using the following inequalities* ${\left\|\hat{X}O_{X}-X^{*}\right\|}_{F}\leq \sqrt{2r}\left\|\hat{X}O_{X}-X^{*}\right\|$ *and* ${\left\|\hat{X}{\hat{X}}^{⊤}-X^{*}{\left(X^{*}\right)}^{⊤}\right\|}_{F}\leq \sqrt{2r}\left\|\hat{X}{\hat{X}}^{⊤}-X^{*}{\left(X^{*}\right)}^{⊤}\right\|\leq 2\sqrt{2r}\left\|\hat{W}-X^{*}{\left(X^{*}\right)}^{⊤}\right\|$*, where the bound of *$\left\|\hat{W}-X^{*}{\left(X^{*}\right)}^{⊤}\right\|$ *is derived in the proof of Theorem 7*.

**Remark 9** *Here we compare our result with the state of art result in matrix completion literature (*Ma et al., 2018*) under the random missing condition. However, we should notice that their theorems don’t hold under the current missing pattern since their entrywise independent sampling assumption is violated. Their operator norm error converges to*

(14) $$\left\|\hat{X}O_{X}-X^{*}\right\|≲\frac{\sigma }{\lambda_{min}(W_{0}^{*})}\sqrt{\frac{N_{0}}{p}}\left\|X^{*}\right\|,$$

*where p is the entrywise sampling probability under their setting. We can show that* $p\approx 1-2{\left(p_{0}-p_{0}^{2}\right)}^{2}/{\left(2p_{0}-p_{0}^{2}\right)}^{2}=\left(2-p_{0}^{2}\right)/{\left(2-p_{0}\right)}^{2}$, $N_{0}\approx N\left(2p_{0}-p_{0}^{2}\right)\approx Np_{0}$, $\lambda_{\text{min}}(W_{0}^{*})\approx p_{0}\lambda_{\text{min}}$ and $\left\|X^{*}\right\|\approx \sqrt{p_{0}r\mu_{0}\lambda_{max}}$ *(see the proof of Theorem 7). As a result, their error bound* (14) *reduces to*

(15) $$\left\|\hat{X}O_{X}-X^{*}\right\|≲\frac{\left(2-p_{0}\right)\sqrt{K}}{\sqrt{\lambda_{min}}}\sqrt{N}\sigma .$$

*When* $p_{0}\to 1$, *our rate is* (12), *which has a difference with* (15) *in the order of* $\sqrt{K}$; *when* $p_{0}\to 0$, *our rate is* (12) *or* (13), *which has the difference with* (15) *in the order of* $K^{5/2}max\left\{1,p_{0}logN\right\}$. *It means that our rate is the same as theirs up to some constants or log factors, which means that the error bound can be similar even under different sampling scenarios. The additional factor K may be caused by the dependence of the sampling pattern*.

**Remark 10** *The minimax lower bound for matrix completion has been established under the random missing setting (*Candès and Tao, 2010; Koltchinskii et al., 2011; Lounici, 2011; Negahban and Wainwright, 2012). *For example, the lower bound for* ${inf}_{{\hat{W}}_{0}}{sup}_{W_{0}^{*}\in 𝒲}\|{\hat{W}}_{0}-W_{0}^{*}{\|}_{2}$ *is* $\sigma \sqrt{N_{0}r/p}$ *(*Lounici, 2011, *Theorem* 3*), where* $𝒲=\{W_{0}^{*}\in R^{N_{0}\times N_{0}}:{\left\|W_{0}\right\|}_{\infty }\leq a,rank(W_{0}^{*})\leq r\}$, $\sigma^{2}$ *is the variance of the Gaussian noise in the observations and p is their entry-wise sampling probability. If the rate is adapted to our setting, it can be rewritten as* $\sqrt{Np_{0}r}\left(2-p_{0}\right)\sigma$ *following the analysis of Remark 9. While in the proof of Theorem 7, we also show that* $\hat{W}$ *defined in* (9) *satisfies* ${\left\|\hat{W}-W_{0}^{*}\right\|}_{2}≲\sqrt{Np_{0}}\left(2-p_{0}\right)K^{2}\sigma$. *Thus, our upper bound matches the minimax rate with regard to the sample size N and the sampling probability* $p_{0}$, *with a difference of the factor* $K^{2}/\sqrt{r}=r^{3/2}\mu_{0}^{2}\tau^{2}$.

**Remark 11** *The sampling* model (1) *assumes independence within each source. Under certain models of dependence, our theoretical results remain valid, as discussed in* Supplementary S.5.

Based on Theorem 7, we generalize it to m>2 sources and derive the following theorem.

**Theorem 12** *Given* 0<ϵ<1, *let* $m=\left\lceil log\epsilon /log\left(1-p_{0}\right)\right\rceil$. *Under Assumptions 1, 2, 3, and 4, with probability at least* $1-O\left(\frac{{log}^{2}\epsilon }{{log}^{2}\left(1-p_{0}\right)N^{3}}\right)$, *we have* n≥(1−ϵ)N *and there exists* $O_{X}\in {𝒪}^{r\times r}$ *such that*

- *if* $p_{0}=o(1/logN)$ *or* $p_{0}$ *is bounded away from* 0, *we have*
(16) $$\left\|\hat{X}O_{X}-X^{*}\right\|≲\left\{1+\frac{\left(1-p_{0}\right)K^{2}{log}^{2}\epsilon }{{log}^{2}\left(1-p_{0}\right)}\sqrt{\frac{p_{0}}{1-{\left(1-p_{0}\right)}^{m}}}\right\}K\sqrt{\frac{N_{0}}{\lambda_{min}}}\sigma ;$$
- otherwise,
(17) $$\left\|\hat{X}O_{X}-X^{*}\right\|≲\left\{1+\frac{\left(1-p_{0}\right)K^{2}\left(p_{0}log\;N\right){log}^{2}\epsilon }{{log}^{2}\left(1-p_{0}\right)}\sqrt{\frac{p_{0}}{1-{\left(1-p_{0}\right)}^{m}}}\right\}K\sqrt{\frac{N_{0}}{\lambda_{min}}}\sigma .$$

**Remark 13** *The above theorem gives us guidance on how many sources we need to recover enough parts of* $W^{*}$. *The order of m can be* $\left|1/log\left(1-p_{0}\right)\right|\approx 1/p_{0}$ *when* $p_{0}$ *is small. Besides, compared to* (12) *and* (13), *the rates of* (16) *and* (17) *have only difference in the log terms, which means that even we choose m of the maximum order above, the rate of our error bounds will not change too much*.

**Remark 14** *The multi-source embeddings have diverse applications, for example, the machine translation (*Mikolov et al., 2013b; Xing et al., 2015; Shi et al., 2020*) or code mapping (*Hernandez et al., 2009; Zhou et al., 2012; Fidahussein and Vreeman, 2014*). To be specific, for each pair of sources* (s,k), *we can match the entity* $i\in {𝒱}_{s}\backslash {𝒱}_{k}$ *to some entity* $j\in {𝒱}_{k}$ *such that*

$$j=arg\;\underset{l\in {𝒱}_{k}}{max}\;cos({\hat{X}}_{i},{\hat{X}}_{l})\;\mathrm{where}\;cos({\hat{X}}_{i},{\hat{X}}_{l})={\left({\hat{X}}_{i}\right)}^{⊤}{\hat{X}}_{l}/(\left\|{\hat{X}}_{i}\right\|\left\|{\hat{X}}_{l}\right\|).$$

*If* $cos({\hat{X}}_{i},{\hat{X}}_{j})$ *is larger than a threshold* *c*, *we can translate the entity i from the sth source (language) to the entity j in the kth source (language). We can determine c by either setting a desired sensitivity using test data or through cross-validation with translated pairs or a specificity that can be approximated by the distribution of cosine similarity of related but not synonymous pairs. Once we obtain the spectral error bound of* $\left\|\hat{X}O_{X}-X^{*}\right\|$, *we can utilize it to construct the bound of the translation accuracy. For example, we can bound* $\left\|{\hat{X}}_{i}-{\hat{X}}_{j}\right\|$ *when* $X_{i}^{*}=X_{j}^{*}$ *or* $P(cos({\hat{X}}_{i},{\hat{X}}_{j})\geq c)$ *when* $cos(X_{i}^{*},X_{j}^{*})\leq c_{0}$ *for some* $c_{0}$. *According to the translation procedure, the translation accuracy can depend on the bound* ${sup}_{l\in {𝒱}_{k}}\left|cos({\hat{X}}_{i},{\hat{X}}_{l})-cos(X_{i}^{*},X_{l}^{*})\right|$ *where* $i\in {𝒱}_{s}\backslash {𝒱}_{k}$. *To bound the quantity, we need additional assumptions on the structures of the underlying matrix* $W^{*}$. *For instance, if the entities i and j are synonym or translated pairs in different languages, then* $cos(X_{j}^{*},X_{i}^{*})>c_{1}$ *for some constant* $c_{1}$, *otherwise*, $cos(X_{j}^{*},X_{i}^{*})\leq c_{0}$ *for some* $c_{0}<c_{1}$.

## 4. Simulation

In this section, we examine the performance of Algorithm 1 from extensive simulation studies for various values of $p_{0}$, *m*, and σ.

### 4.1 Comparable Methods

We compare with SMC (Cai et al., 2016) and a state-of-the-art matrix completion algorithm under the uniform sampling assumption, vanilla gradient descent (VGD) (Ma et al., 2018). Since SMC can only be applied to complete a single missing block, we use it to complete the missing blocks of each pair of sources. After all missing blocks are imputed, we use the rank-*r* SVD to obtain the low-rank estimator for SMC. For VGD, the input is the partially observed matrix $\tilde{W}$ created in Step I (b) of Algorithm 1, where the unobserved entries are treated as missing values. They will also produce an estimator for $X^{*}$. Another standard approach is to use one data source as pre-training and the new data sources to continue training. This effectively corresponds to imputing the missing blocks of the PMI matrix as zero. We call the method ‘Pre-trained’.

Besides, a potential application of BONMI is machine translation. To be specific, in reality, the overlapping parts may not be known fully. For instance, ${\left\{W^{s}\right\}}_{s\in [m]}$ are multilingual co-occurrence matrices or PMI matrices (Levy and Goldberg, 2014), then each vertex is a word and the overlapping parts are created by bilingual dictionaries, which are limited in some low-resource languages and always cover only a small proportion of the corpora. In this case, BONMI can utilize these matrices and their known overlap to train multilingual word embeddings (i.e., $\hat{X}$). For the words not known in the overlapping set, if their embeddings (i.e., rows of $\hat{X}$) are close enough, it means that they have a similar meaning and should be translated to each other. We evaluate the translation precision in the simulation setting (iii). As a baseline, we also compare BONMI to the popular orthogonal transformation method (Smith et al., 2017) which uses the single-source embeddings ${\hat{X}}_{s}={\hat{U}}_{s}{\hat{\Sigma }}_{s}^{1/2}$ for s∈[m]. We denote the method as ‘Orth’.

For all methods, we use the true rank *r* for simplicity in the following numeric experiments. We propose a data-driven method for choosing *r* for real-world problems in Section 5. To validate this proposed tuning strategy for *r*, we evaluate the performance of all algorithms using the estimated *r* in simulations. As summarized in Supplementary S.6, the relative performance of different algorithms shares a similar pattern as those given in Section 4.3 with BONMI outperforming other competing methods.

### 4.2 Data Generation Mechanisms and Evaluation Metrics

Throughout, we fix *N* = 25,000 and *r* = 200 which are comparable to our real data. We then generate the word embedding matrix $X=U^{*}{\left(\Sigma^{*}\right)}^{\frac{1}{2}}$ and therefore $W^{*}=U^{*}\Sigma^{*}{\left(U^{*}\right)}^{⊤}$, where $\Sigma^{*}$ is a diagonal matrix whose diagonal elements are generated independently from the uniform distribution $U(\sqrt{N},4\sqrt{N})$. The matrix $U^{*}$ is drawn randomly from the Haar measure. Specifically, we generate a matrix $H\in R^{N\times r}$ with i.i.d. standard Gaussian entries, then apply the QR decomposition to **H** and assign $U^{*}$ with the Q part of the result. To generate data for the *s*th source, we generate a sequence of independent Bernoulli random variables with success rate $p_{0}:\delta^{s}=\left(\delta_{1}^{s},\ldots ,\delta_{N}^{s}\right)$ to form the index set ${𝒱}_{s}=\left\{i:\delta_{i}^{s}=\right.1,i\in [N]\}$, for s∈[m]. We then generate the error matrix $E^{s}\in R^{\left|{𝒱}_{s}\right|\times \left|{𝒱}_{s}\right|}$ with its upper triangular block including the diagonal elements from the normal distribution $N\left(0,\sigma_{s}^{2}\right)$ and lower triangular block decided by symmetry, where we let the noise level $\sigma_{s}$ vary across the *m* sources.

We consider three settings with the first two focusing on the task of matrix completion and setting (iii) focusing on the downstream task of machine translation. For the matrix completion task, we consider two settings: (i) m=2, $\sigma_{s}=0.1s$, and let $p_{0}$ vary from 0.1 to 0.3 ; (ii) $p_{0}=0.1$, $\sigma_{s}=0.1$, and let *m* vary from 2 to 6. For the machine translation task, we consider the setting (iii) where we let m=2,3, $p_{0}=0.1$, $\sigma_{s}=s\sigma$ and let a noise level σ vary from 0.3 to 0.5.

To evaluate the performance of matrix completion, we use the relative F-norm and spectral norm errors of the estimation of $W_{0}^{*}$ defined as ${err}_{F}(\hat{W},W_{0}^{*})=\frac{{\left\|\hat{W}-W_{0}^{*}\right\|}_{F}}{{\left\|W_{0}^{*}\right\|}_{F}}$ and ${err}_{2}(\hat{W},W_{0}^{*})=\frac{\left\|\hat{W}-W_{0}^{*}\right\|}{\left\|W_{0}^{*}\right\|}$. To evaluate the overall performance of machine translation in setting (iii), we additionally generate test data for evaluation. Specifically, we additionally sample $n_{\text{test}}=2000$ vertices from $𝒱\backslash {𝒱}^{*}$ where $𝒱*=\cup_{s=1}^{m}{𝒱}_{s}$, denoted as ${𝒱}^{\text{test}}$, and combine ${𝒱}^{\text{test}}$ and ${𝒱}_{s}$ to get ${𝒱}_{s}^{\prime }={𝒱}^{\text{test}}\cup {𝒱}_{s}$ as the final vertex set of the *s*th source. We then use ${𝒱}_{s}^{\prime }$ to generate $W^{s}$. Notice now $E^{s}\in R^{\left|{𝒱}_{s}^{\prime }\right|\times \left|{𝒱}_{s}^{\prime }\right|}$. However, we treat elements of ${𝒱}^{\text{test}}$ as unique across the *m* sources, which means that we will not combine elements of ${𝒱}^{\text{test}}$ in Algorithm 1. The role of ${𝒱}^{\text{test}}$ is exactly the testing set in machine translation. We average the m−1 translation precision from the *s*th source to the 1st source, s=2,…,m. The translation precision is defined as follows: for $i\in {𝒱}^{\text{test}}$, we can get its embedding in the *s*th source corresponding to one row in $\hat{X}$, denoted as ${\hat{X}}_{i}$. Then we find its closest vector ${\hat{X}}_{j}$ for $j\in {𝒱}_{1}^{\prime }$ with the largest cosine similarity as illustrated in Remark 14. If the *j*th element from the 1st source and the *i*th element from the *s*th source are the same element in ${𝒱}^{*}$, we treat it as a correct translation. The precision of the *s*th source is the ratio of correct translations among the test set ${𝒱}^{\text{test}}$ in the *s*th source.

### 4.3 Results

**Figure 1:**
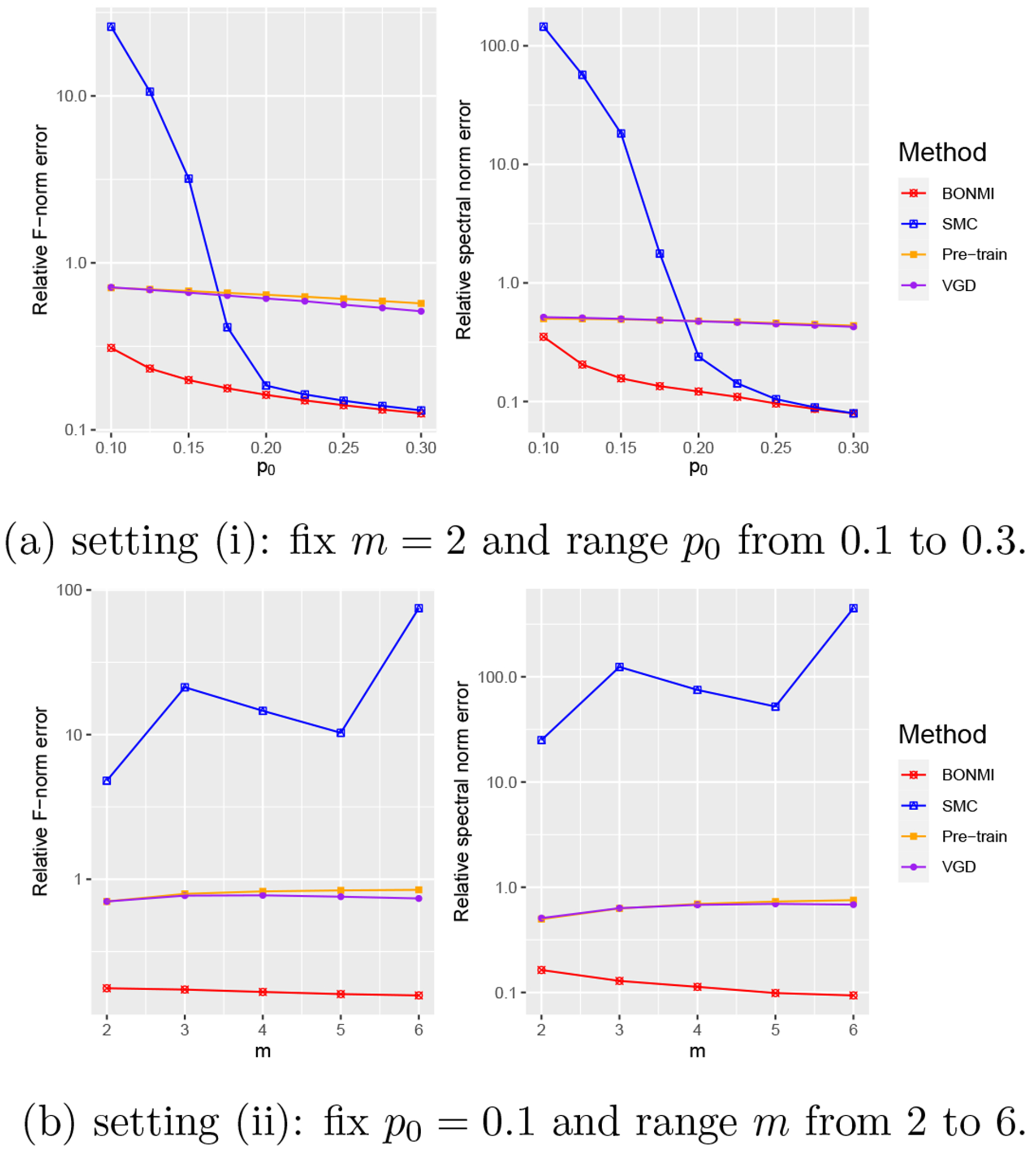
Simulation results of settings (i) and (ii). The relative estimation errors of $W_{0}^{*}$ are presented.

**Figure 2:**
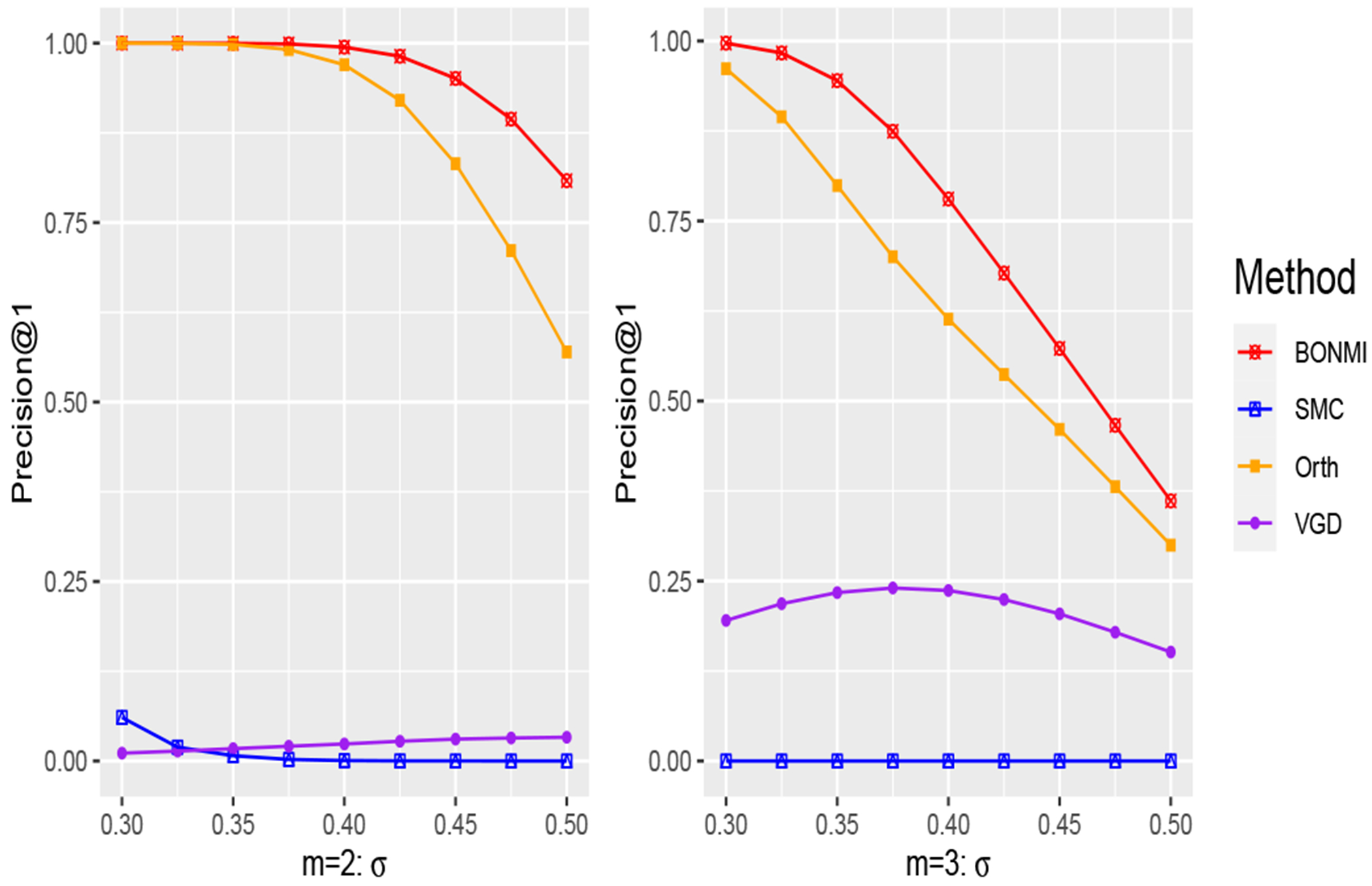
setting (iii): fix *p*_0_ = 0.1 and range *σ* from 0.3 to 0.5.

We summarize simulation results averaged over 50 replications for settings (i)-(ii) in Figure 1 and setting (iii) in Figure 2. BONMI outperforms all competing methods across the three settings. In settings (i) and (ii), the results of the F-norm and spectral norm errors are consistent. In setting (i), we can see that the relative errors of all methods decrease when the observation rate *p*_0_ increases as expected. The advantage of BONMI in the accuracy of matrix completion is more pronounced when the observation rate *p*_0_ is low. When *p*_0_ is very small, SMC tends to fail. In setting (ii), the error of BONMI decreases as *m* increases, which is due to the information gained from multiple sources. However, both the naive pretraining method and VGD do not always perform better as *m* increases. Overall, BONMI dominates all competing methods across different choices of *m*.

## 5. Real Data Analysis

In this section, we apply BONMI to obtain clinical concept embeddings using multiple PPMI matrices in two different languages, English and Chinese. The clinical concepts in English have been mapped to *Concept Unique Identifiers* (CUIs) in the Unified Medical Language System (UMLS) (Humphreys and Lindberg, 1993). Our goal is to enable the integration of multiple PPMI matrices to co-train clinical concept embeddings for both CUIs and Chinese clinical terms.

The input data ensemble consists of three CUI PPMI matrices and one Chinese PPMI matrix. The three CUI PPMI matrices are independently derived from three data sources (i) 20 million clinical notes at Stanford (Finlayson et al., 2014); (ii) 10 million notes of 62K patients at Partners Healthcare System (PHS) (Beam et al., 2019); and (iii) health records from MIMIC-III, a freely accessible critical care database (Johnson et al., 2016). The multi-source raw data consist of the text data (i) and the EHR data (ii) and (ii), which are prepossessed in the same way as Beam et al. (2019) and Hong et al. (2021) to generate the CUI-level co-occurrence matrices. The PPMI matrices are then obtained following Section 2.2. We choose sub-matrices from these sources by thresholding the frequency of these CUI and keeping those with semantic types related to medical concepts. Finally, we obtain the Stanford, PHS, and MIMIC PPMI matrices with 8922, 10964, and 8524 CUIs respectively. The mean overlapping CUIs of any two sources is 4480 and the total number of the unique CUIs of the three sources is 17963.

**Figure 3:**
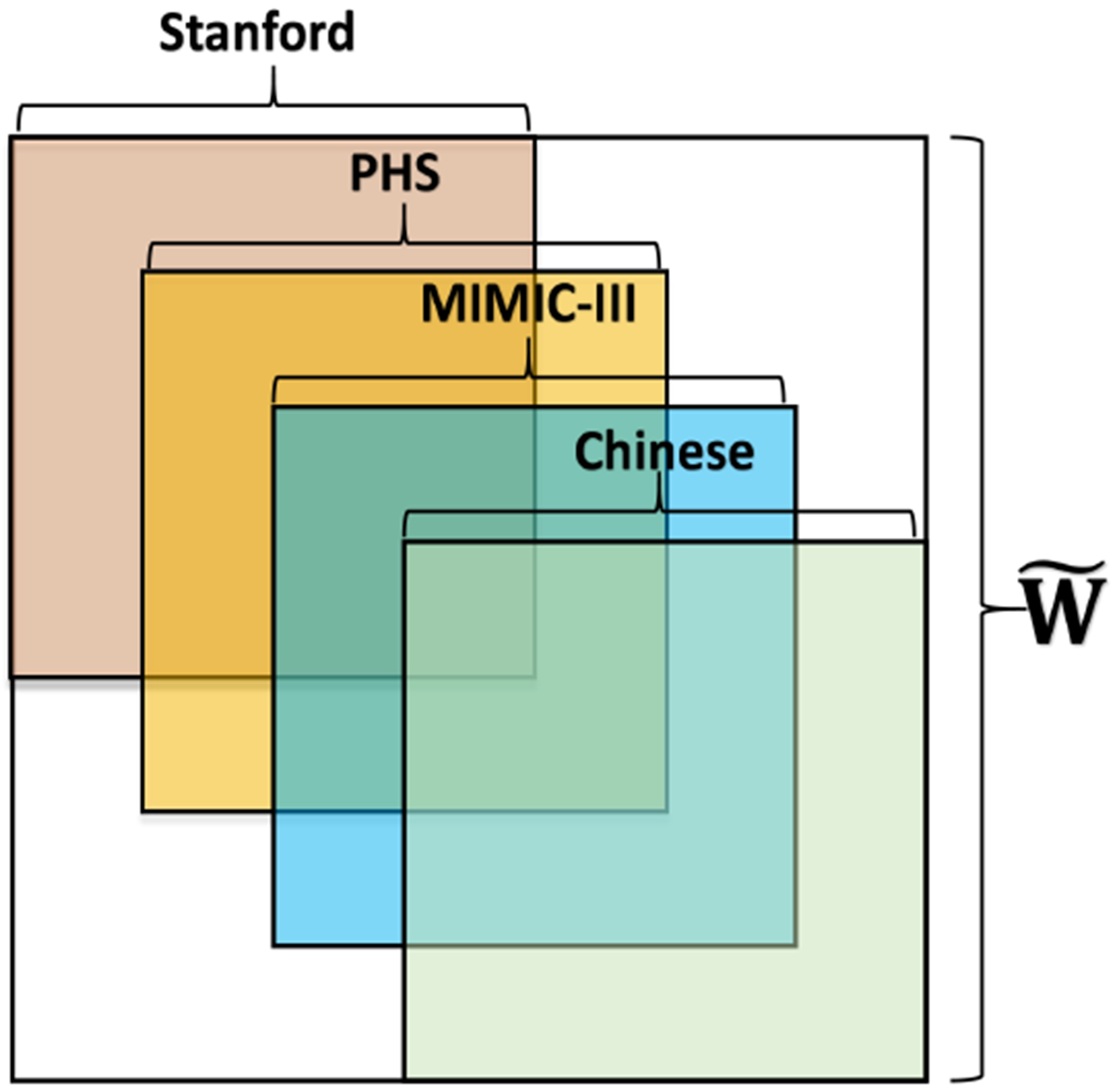
The aggregation of the four PPMI matrices. The four sources all have an overlapping with each other source.

Multiple Chinese medical text data sources, such as medical textbooks and Wikipedia, are also collected. We then build a PPMI matrix of 8628 Chinese medical terms. A Chinese-English medical dictionary is used to translate these Chinese medical terms to English, which are further mapped to CUI. Finally, we obtain 4201 Chinese-CUI pairs, and we use 2000 pairs as the training set (the known overlapping set) and the other 2201 pairs as the test set to evaluate the translation precision. An illustration of the aggregation of the three CUI PPMI matrices and one Chinese PPMI matrix is presented in Figure 3.

From each method, we obtain the embedding vectors for all entities by performing an SVD on the imputed PPMI matrix obtained from each method, respectively. To evaluate the quality of the obtained embedding, we compare the cosine similarity of trained embeddings against the gold standard human annotations of the concept similarity and relatedness. We considered two sets of human annotated relatedness and similarity: (I) 200 pairs of Chinese medical terms randomly selected and annotated by four clinical experts; and (II) 566 pairs of UMLS concepts in English previously annotated by eight researchers in Pakhomov et al. (2010). The Chinese medical terms were also translated into English and mapped to the UMLS CUI while the 566 UMLS concepts in English were also translated into Chinese. Each concept pair thus can be viewed as CUI-CUI pair (CUI), Chinese-Chinese pair (Chinese), and Chinese-CUI pair (Cross). The gold standard human annotation assigns each concept pair a relatedness and similarity score, defined as the average score from all reviewers. For each concept pair, we compare the cosine similarity of their associated embeddings against the human annotations of their similarity and relatedness. We evaluate the quality of the embeddings based on (a) the rank correlation between the cosine similarity and human annotation; and (b) the accuracy in translating Chinese medical terms to CUIs in English. The Precision@*k* is defined similarly to the translation accuracy in Section 4. The difference is that when the truth CUI is among the CUIs with the top *k* largest cosine similarity to the Chinese term, then it is treated as a correct translation. Precision@*k* is the ratio of the correct translations given a *k*. Here we choose *k* as 5, 10 and 20.

To choose the rank of the matrix, we analyze the eigen decay of the matrices. The eigen decay has been widely used to determine the rank of low-rank matrices, for example, in principal component analysis (Jolliffe, 2005), word embedding (Hong et al., 2021) and network analysis (Arroyo et al., 2021). We calculate the eigen decay of the overlapping sub-matrices of each pair of sources and choose the rank *r* that makes the cumulative eigenvalue percentage of at least one of the matrices more than 95%, which is 300. We then use *r* = 300 for all methods.

Finally, to compare with the neural-based embeddings, we also include a BERT-based algorithm CODER (mediCal knOwledge embeDded tErm Representation) proposed by Yuan et al. (2022). The CODER algorithm is trained on top of BERT (Devlin et al., 2019) with contrastive learning on the multi-lingual relation pairs from UMLS (Bodenreider, 2004) to improve medical term embedding. We use the pre-trained model of Yuan et al. (2022), whose input is the code descriptions, where we use the preferred English terms for CUIs and Chinese terms, and the output of CODER is the CUI and Chinese embeddings of dimension 768.

Besides, the machine translation accuracy can suffer from the limited size of the Chinese corpus. On the other hand, CODER embeddings utilize the multi-lingual semantic information from code descriptions, which serves as complementary information to the PMI-based embeddings. As a result, we use the CODER embeddings to assist the machine translation task by concatenating their embeddings with the EHR-based embeddings (denoted by ‘method+’, e.g., ‘BONMI+’).

### 5.1 Results

We present the results of BONMI and other competing methods in Table 1. We can observe that all methods other than SMC perform similarly when assessing the relatedness and similarity of Chinese-Chinese and CUI-CUI since these pairs belong to the same corpus. However, BONMI outperforms other methods when evaluating relatedness and similarity between Chinese-CUI pairs that belong to the missing blocks. BONMI also attains higher accuracy in the translation task. This suggests that BONMI has the advantage over existing methods in providing embedding vectors that enable an accurate assessment of relatedness between entity pairs that do not belong to the same corpus.

## 6. Discussion and Conclusion

### 6.1 Generalization

We consider the completion of the PMI matrices and the estimation of multi-source embeddings in this paper. However, we also notice that there exist many applications involving the completion of missing blocks for asymmetric matrices, for example, genomic data integration (Cai et al., 2016), multimodality data analysis (Xue and Qu, 2020), and other applications mentioned in the introduction. Hence, in this section, we introduce an algorithm designed for asymmetric matrices without repeated observations. Now assume that $W^{*}\in R^{N\times D}$ of rank *r* is asymmetric with $\lambda_{max}=\sigma_{1}(W^{*})>\lambda_{min}=\sigma_{r}(W^{*})>0$. Here $W^{*}$ does not have to come from the inner product of word embeddings but can be any low-rank matrix. Furthermore, we have a noisy-corrupted matrix **W** such that $W=W^{*}+E$. For the *s*th source, we sample two index sets ${𝒱}_{s1}\subseteq [N]$ and ${𝒱}_{s2}\subseteq [D]$ independently such that for each i∈[N] and j∈[D], we assign *i* to ${𝒱}_{s1}$ with probability $p_{s1}$ and *j* to ${𝒱}_{s2}$ with probability $p_{s2}$ independently:

$$P\left(i\in {𝒱}_{s1}\right)=p_{s1}\in (0,1),\;\text{for}\;i\in [N],\;P\left(j\in {𝒱}_{s2}\right)=p_{s2}\in (0,1),\;\text{for}\;j\in [D],s\in [m].$$

With the index sets ${𝒱}_{s1}$ and ${𝒱}_{s2}$, a matrix $W^{s}$ is observed

(18) $$W^{s}=W_{{𝒱}_{s1},{𝒱}_{s2}},\;\text{for}\;s\in \left[m\right].$$

Let ${𝒱}_{1}^{*}=\cup_{s=1}^{m}{𝒱}_{s1}$ and ${𝒱}_{2}^{*}=\cup_{s=1}^{m}{𝒱}_{s2}$, then our task is to recover

$$W_{0}^{*}\equiv W_{{𝒱}_{1}^{*},{𝒱}_{2}^{*}}^{*}={\left[W^{*}(i,j)\right]}_{j\in {𝒱}_{2}^{*}}^{i\in {𝒱}_{1}^{*}}\in R^{n_{1}\times n_{2}},\;\text{where}\;N_{0}=\left|{𝒱}_{1}^{*}\right|\;\text{and}\;D_{0}=\left|{𝒱}_{2}^{*}\right|.$$

Without loss of generality, we assume ${𝒱}_{1}^{*}=\left[N_{0}\right]$ and ${𝒱}_{2}^{*}=\left[D_{0}\right]$. The estimation procedure is summarized in Algorithm 2.

The model (18) is more general than (2) in two ways: (i) (18) considers the asymmetric matrix and (ii) (18) does not assume repeated observations. To be specific, the overlapping parts of $W^{s}$ are the same for different sources, which means that they are only observed once, and Step I Aggregation in Algorithm 1 is not applicable now. The two relaxations make the model (18) more flexible and realistic for the applications mentioned above. However, if one does have repeated observations from each source with the heterogeneous noise level, a weighted aggregation procedure similar to Step I (b) of Algorithm 1 can be applied and will not affect the theoretical guarantee of the estimator.

Assume that $W_{0}^{*}$ has SVD $W_{0}^{*}=U_{0}^{*}\Sigma_{0}^{*}{\left(V_{0}^{*}\right)}^{⊤}=X^{*}{\left(Y^{*}\right)}^{⊤}$, where $U_{0}^{*}\in R^{N_{0}\times r}$ are the left-singular vectors, $V_{0}^{*}\in R^{D_{0}\times r}$ are the right-singular vectors, and $\Sigma_{0}^{*}$ is an r×r diagonal matrix with singular values in a descending order. In addition, $X^{*}=U_{0}^{*}{\left(\Sigma_{0}^{*}\right)}^{1/2}\in R^{N_{0}\times r}$ and $Y^{*}=V_{0}^{*}{\left(\Sigma_{0}^{*}\right)}^{1/2}\in R^{D_{0}\times r}$. Let $\hat{X}=\hat{U}{\hat{\Sigma }}^{1/2}$ and $\hat{Y}=\hat{V}{\hat{\Sigma }}^{1/2}$ be the output of Algorithm 2. Without loss of generality, assume N=max{N,D} and $p_{0}={min}_{s\in [m]}\left\{p_{s1},p_{s2}\right\}$. Similar results to Theorem 7 and Theorem 12 can be provided. For example, when m=2, if $p_{0}=o(1/log\;N)$ or $p_{0}$ is bounded away from zero, we can prove that under similar assumptions,


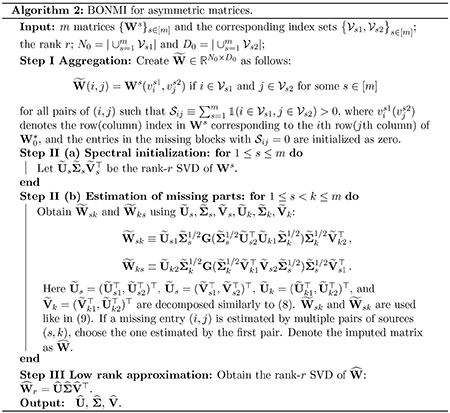



$$\left\|\hat{X}O_{X}-X^{*}\right\|≲\frac{\left\{\left(1-p_{0}\right)K^{2}+1\right\}K}{\sqrt{\lambda_{min}}}\sqrt{N}\sigma ,\;\left\|\hat{Y}O_{Y}-Y^{*}\right\|≲\frac{\left\{\left(1-p_{0}\right)K^{2}+1\right\}K}{\sqrt{\lambda_{min}}}\sqrt{N}\sigma ,$$

and otherwise,

$$\left\|\hat{X}O_{X}-X^{*}\right\|≲\frac{\left\{\left(1-p_{0}\right)K^{2}\left(p_{0}\log N\right)+1\right\}K}{\sqrt{\lambda_{min}}}\sqrt{N}\sigma ,$$

$$\left\|\hat{Y}O_{Y}-Y^{*}\right\|≲\frac{\left\{\left(1-p_{0}\right)K^{2}\left(p_{0}\log N\right)+1\right\}K}{\sqrt{\lambda_{min}}}\sqrt{N}\sigma ,$$

for some rotation matrices $O_{X},O_{Y}\in {𝒪}^{r\times r}$. The proofs are the simple extensions of the proof of Theorem 7 and Theorem 12.

### 6.2 Conclusion

This paper proposes BONMI, which aims at multi-source learning. Our method is computationally efficient with a theoretical guarantee. The performance of our algorithm is verified by simulation and real data analysis. For theoretical guarantee, we require the sampling probability of each source to have the order of $\sqrt{log\;N/N}$, which is a small order of *N* and can be satisfied easily in many applications. Besides, we extend BONMI to asymmetric matrices without repeated observations for other potential applications such as genomic data integration.

## Supplementary Material

1

## Figures and Tables

**Table 1:** Results of the integration of four PPMI matrices: (a) rank correlation between the pairwise cosine similarity of estimated embedding vectors from the completed matrix and the similarity or relatedness from human annotation; (b) accuracy in translation based on the estimated embedding vectors.

(a) Rank correlation with human annotations							
Source	Type	Set	BONMI	Pre-train	SMC	VGD	CODER
Chinese	Rel	I	0.741	0.756	0.066	0.761	0.519
	Rel	II	0.661	0.659	0.327	0.663	0.482
	Sim	I	0.707	0.724	0.105	0.731	0.715
	Sim	II	0.716	0.728	0.271	0.726	0.469

CUI	Rel	I	0.678	0.639	0.369	0.643	0.398
	Rel	II	0.604	0.598	0.141	0.592	0.351
	Sim	I	0.615	0.601	0.243	0.582	0.741
	Sim	II	0.634	0.635	0.171	0.622	0.451

Cross	Rel	I	0.671	0.408	0.321	0.418	0.502
	Rel	II	0.655	0.424	0.301	0.358	0.424
	Sim	I	0.607	0.322	0.369	0.339	0.724
	Sim	II	0.699	0.445	0.335	0.399	0.428

(b) Translation accuracy						
		BONMI+	Pre-train+	SMC+	VGD+	CODER
Precision	@5	0.708	0.617	0.569	0.614	0.553
	@10	0.766	0.683	0.633	0.677	0.621
	@20	0.812	0.728	0.691	0.719	0.683
