# Supplementary material for: Multi-source Learning via Completion of Block-wise Overlapping Noisy Matrices
Source: J Mach Learn Res. Author manuscript; Available in PMC 2026 Mar 27. (PMC13021262)
Supplement: 1 [file NIHMS2120565-supplement-1.pdf]

Technical supplementary material to

Multi-source Learning via Completion of Block-wise Overlapping Noisy Matrices

This document contains the supplementary material to the paper "Multi-source Learning via Completion of Block-wise Overlapping Noisy Matrices". We mainly provide technical details of proving Proposition 2, Theorems 7, and 12 here.

S.1. Proof of Proposition 2

Proof First, we prove $\mathrm{rank}(\mathbf{W}^*_{s\cap k,s\cap k}) = r$. Let $N_{sk} \equiv |\mathcal{V}_s \cap \mathcal{V}_k|$, then by Lemma S.1, we have

$$N_{sk} \geq \frac{3p_s p_k N}{2} \geq C\mu_0 r \log N$$

holds with probability $1 - O(1/N^3)$ for some sufficiently large constant C, where the second inequality comes from Assumption 2. Then, by Lemma S.2, we have

$$\lambda_r(\mathbf{W}^*_{s\cap k,s\cap k}) \geq \sigma_{\min}(\mathbf{U}^*_{s\cap k})\lambda_r(\mathbf{\Sigma}^*)\sigma_{\min}(\mathbf{U}^*_{s\cap k}) \geq \frac{n_{sk}\lambda_{\min}}{2N} > 0$$

with probability $1 - 1/N^3$. As a result, $\mathrm{rank}(\mathbf{W}^*_{s\cap k,s\cap k}) = r$ with probability as least $1 - O(1/N^3)$. Under this event, since $\mathbf{W}^*_{s\cap k,s\cap k}$ is a principal sub-matrix of $\mathbf{W}^*_s$, we have $\mathrm{rank}(\mathbf{W}^*_s) \geq \mathrm{rank}(\mathbf{W}^*_{s\cap k,s\cap k}) = r$. Besides, $\mathbf{W}^*_s$ is a principal sub-matrix of $\mathbf{W}^*$, we have $\mathrm{rank}(\mathbf{W}^*_s) \leq \mathrm{rank}(\mathbf{W}^*) = r$. Combining the two inequalities, we have $\mathrm{rank}(\mathbf{W}^*_s) = r$. The same conclusion holds for $\mathbf{W}^*_k$. We then prove that $\mathbf{W}^*_{s\setminus k,k\setminus s}$ has the representation of (6). Recall the eigen-decomposition of $\mathbf{W}^* = \mathbf{U}^*\mathbf{\Sigma}^*(\mathbf{U}^*)^\top$ and by definition we will have

$$\mathbf{W}^*_{s\cap k,s\cap k} = \mathbf{U}^*_{s\cap k}\mathbf{\Sigma}^*(\mathbf{U}^*_{s\cap k})^\top = \mathbf{V}^*_{s2}\mathbf{\Sigma}^*_s(\mathbf{V}^*_{s2})^\top = \mathbf{V}^*_{k1}\mathbf{\Sigma}^*_k(\mathbf{V}^*_{k1})^\top, \tag{S.1}$$

which also implies that $\mathrm{rank}(\mathbf{V}^*_{s2}) = \mathrm{rank}(\mathbf{V}^*_{k1}) = r$. Multiplying $\mathbf{V}^*_{k1}$ on the both sides of the last equation, we obtain

$$\mathbf{V}^*_{s2}(\mathbf{\Sigma}^*_s)^{1/2}(\mathbf{\Sigma}^*_s)^{1/2}(\mathbf{V}^*_{s2})^\top\mathbf{V}^*_{k1} = \mathbf{V}^*_{k1}(\mathbf{\Sigma}^*_k)^{1/2}(\mathbf{\Sigma}^*_k)^{1/2}(\mathbf{V}^*_{k1})^\top\mathbf{V}^*_{k1}$$

and the following equation

$$\mathbf{V}^*_{k1}(\mathbf{\Sigma}^*_k)^{1/2} = \mathbf{V}^*_{s2}(\mathbf{\Sigma}^*_s)^{1/2}\widehat{\mathbf{R}},$$

where $\widehat{\mathbf{R}} = (\mathbf{\Sigma}^*_s)^{1/2}(\mathbf{V}^*_{s2})^\top\mathbf{V}^*_{k1}\big((\mathbf{V}^*_{k1})^\top\mathbf{V}^*_{k1}\big)^{-1}(\mathbf{\Sigma}^*_k)^{-1/2}$. It is easy to verify that $\widehat{\mathbf{R}}^\top\widehat{\mathbf{R}} = \mathbf{I}_r$ and then it is obvious that

$$\widehat{\mathbf{R}} = \arg\min_{\mathbf{R}\in\mathscr{O}^{r\times r}} \|\mathbf{V}^*_{s2}(\mathbf{\Sigma}^*_s)^{1/2}\mathbf{R} - \mathbf{V}^*_{k1}(\mathbf{\Sigma}^*_k)^{1/2}\|_{\mathrm{F}}.$$

Then by Lemma 22 of Ma et al. (2018), we prove that $\widehat{\mathbf{R}} = \mathbf{G}\big((\mathbf{V}^*_{s2}(\mathbf{\Sigma}^*_s)^{1/2})^\top\mathbf{V}^*_{k1}(\mathbf{\Sigma}^*_k)^{1/2}\big)$.

Again by (S.1), we have

$$(\mathbf{U}^*_{s\cap k})^\top = \mathbf{\Sigma}^{*-1}\big((\mathbf{U}^*_{s\cap k})^\top\mathbf{U}^*_{s\cap k}\big)^{-1}(\mathbf{U}^*_{s\cap k})^\top\mathbf{V}^*_{k1}\mathbf{\Sigma}^*_k(\mathbf{V}^*_{k1})^\top. \tag{S.2}$$

In addition, we have

$$\mathbf{U}^*_{s\cap k}\mathbf{\Sigma}^*(\mathbf{U}_{s\setminus k})^\top = \mathbf{W}^*_{s\cap k,k\setminus s} = \mathbf{V}^*_{k1}\mathbf{\Sigma}^*_k(\mathbf{V}^*_{k2})^\top. \tag{S.3}$$

Combining (S.2) and (S.3), we have

$$\begin{aligned}
&(\mathbf{U}^*_{s\cap k})^\top\mathbf{V}^*_{k1}\{(\mathbf{V}^*_{k1})^\top\mathbf{V}^*_{k1}\}^{-1}(\mathbf{V}^*_{k2})^\top\\
&=\mathbf{\Sigma}^{*-1}\big((\mathbf{U}^*_{s\cap k})^\top\mathbf{U}^*_{s\cap k}\big)^{-1}(\mathbf{U}^*_{s\cap k})^\top\mathbf{V}^*_{k1}\mathbf{\Sigma}^*_k(\mathbf{V}^*_{k1})^\top\mathbf{V}^*_{k1}\{(\mathbf{V}^*_{k1})^\top\mathbf{V}^*_{k1}\}^{-1}(\mathbf{V}^*_{k2})^\top\\
&=\mathbf{\Sigma}^{*-1}\big((\mathbf{U}^*_{s\cap k})^\top\mathbf{U}^*_{s\cap k}\big)^{-1}(\mathbf{U}^*_{s\cap k})^\top\mathbf{V}^*_{k1}\mathbf{\Sigma}^*_k(\mathbf{V}^*_{k2})^\top\\
&=\mathbf{\Sigma}^{*-1}\big((\mathbf{U}^*_{s\cap k})^\top\mathbf{U}^*_{s\cap k}\big)^{-1}(\mathbf{U}^*_{s\cap k})^\top\mathbf{U}^*_{s\cap k}\mathbf{\Sigma}^*(\mathbf{U}_{s\setminus k})^\top = (\mathbf{U}_{s\setminus k})^\top,
\end{aligned} \tag{S.4}$$

where the first equation comes from (S.2) and the second equation comes from (S.3). Finally,

$$\begin{aligned}
&\mathbf{V}^*_{s1}(\mathbf{\Sigma}^*_s)^{1/2}\mathbf{G}((\mathbf{\Sigma}^*_s)^{1/2}(\mathbf{V}^*_{s2})^\top\mathbf{V}^*_{k1}(\mathbf{\Sigma}^*_k)^{1/2})(\mathbf{\Sigma}^*_k)^{1/2}(\mathbf{V}^*_{k2})^\top\\
&=\mathbf{V}^*_{s1}(\mathbf{\Sigma}^*_s)^{1/2}(\mathbf{\Sigma}^*_s)^{1/2}(\mathbf{V}^*_{s2})^\top\mathbf{V}^*_{k1}\{(\mathbf{V}^*_{k1})^\top\mathbf{V}^*_{k1}\}^{-1}(\mathbf{\Sigma}^*_k)^{-1/2}(\mathbf{\Sigma}^*_k)^{1/2}(\mathbf{V}^*_{k2})^\top\\
&=\mathbf{U}^*_{s\setminus k}\mathbf{\Sigma}^*(\mathbf{U}^*_{s\cap k})^\top\mathbf{V}^*_{k1}\{(\mathbf{V}^*_{k1})^\top\mathbf{V}^*_{k1}\}^{-1}(\mathbf{V}^*_{k2})^\top\\
&=\mathbf{U}^*_{s\setminus k}\mathbf{\Sigma}^*(\mathbf{U}^*_{k\setminus s})^\top = \mathbf{W}^*_{s\setminus k,k\setminus s},
\end{aligned}$$

where the first equation comes from $\widehat{\mathbf{R}} = \mathbf{G}\big((\mathbf{V}^*_{s2}(\mathbf{\Sigma}^*_s)^{1/2})^\top\mathbf{V}^*_{k1}(\mathbf{\Sigma}^*_k)^{1/2}\big)$, the second equation comes from $\mathbf{V}^*_{s1}\mathbf{\Sigma}^*_s(\mathbf{V}^*_{s2})^\top = \mathbf{W}^*_{s\setminus k,s\cap k} = \mathbf{U}^*_{s\setminus k}\mathbf{\Sigma}^*(\mathbf{U}^*_{s\cap k})^\top$, and the third equation comes from (S.4). Then we finish the proof. ■

S.2. Proof of Theorem 7

When $m = 2$, we adopt the notations of Section 2.4 by assuming the two observed submatrices are $\mathbf{W}^s$ and $\mathbf{W}^k$. To prove the theorem, recall that $\widetilde{\mathbf{W}}_{sk}$ defined by (8) is the estimate of $\mathbf{W}^*_{s\setminus k,k\setminus s}$, the main effort lies on the perturbation bound of $\|\widetilde{\mathbf{W}}_{sk} - \mathbf{W}^*_{s\setminus k,k\setminus s}\|$. After we obtain it, the perturbation bound of $\|\widehat{\mathbf{W}} - \mathbf{W}^*\|$ can also be figured out. As a result, the error of the rank r factorization of $\widehat{\mathbf{W}}$ can also be bounded, which leads to Theorem 7. Before we derive $\|\widetilde{\mathbf{W}}_{sk} - \mathbf{W}^*_{s\setminus k,k\setminus s}\|$, we need the basic spectral properties of $\mathbf{W}^*_0$ defined in (5), $\mathbf{W}^*_s$, $\mathbf{W}^*_k$ defined in (4), which are presented in the Section S.2.1.

S.2.1 Characterization of The Underlying Matrix

Recall that $N_s = |\mathcal{V}_s|$, $N_k = |\mathcal{V}_k|$ and $N_{sk} = |\mathcal{V}_s \cap \mathcal{V}_k|$. First, by Lemma S.1, we have

$$\frac{p_l N}{2} \le N_l \le \frac{3p_l N}{2}, \text{ for } l = s,k \text{ and } \frac{p_s p_k N}{2} \le N_{sk} \le \frac{3p_s p_k N}{2} \tag{S.5}$$

hold simultaneously with probability $1 - O(1/N^3)$. Throughout, our analysis is conditional on (S.5). By Proposition S.3, we have

$$\lambda_r(\mathbf{W}^*_l) \ge \frac{N_l\lambda_{\min}}{2N} \ge \frac{p_l\lambda_{\min}}{4}, \text{ for } l = s,k$$

hold simultaneously with probability $1-2/N^3$ since by the Assumption 2, we have $N_l \geq Np_0/2 \geq 16\mu_0 r(\log r + \log N^3), l = s,k$. Then by Lemma S.4, we will have

$$\mu_l = \mu(\mathbf{V}_l^*) = \frac{N_l}{r}\max_{i=1,\ldots,n_l}\sum_{j=1}^{r}\mathbf{V}_l^*(i,j)^2 \leq 2\tau\mu_0, l = s,k.$$

In addition, by Proposition S.5, we have

$$\lambda_1(\mathbf{W}_l^*) \leq \frac{n_l r\mu_0}{N}\lambda_{\max} \leq \frac{3p_l r\mu_0}{2}\lambda_{\max}, \text{ for } l = s,k.$$

As a result, we have the condition number of $\mathbf{W}_l^*$:

$$\tau_l = \lambda_1(\mathbf{W}_l^*)/\lambda_r(\mathbf{W}_l^*) \leq 6r\mu_0\tau, \text{ for } l = s,k.$$

S.2.2 Imputation Error

After we characterize the spectral properties of $\mathbf{W}_0^*$ defined in (5), $\mathbf{W}_l^*, l = s,k$, we begin to control $\|\widetilde{\mathbf{W}}_{sk} - \mathbf{W}^*_{s\backslash k,k\backslash s}\|$. Using the notations of Proposition 2 and Section 2.4, we define

$$\begin{aligned}
&\mathbf{A} = \mathbf{V}_s^*(\mathbf{\Sigma}_s^*)^{1/2};\ \mathbf{B} = \mathbf{V}_k^*(\mathbf{\Sigma}_k^*)^{1/2};\ \widetilde{\mathbf{A}} = \widetilde{\mathbf{V}}_s(\widetilde{\mathbf{\Sigma}}_s)^{1/2};\ \widetilde{\mathbf{B}} = \widetilde{\mathbf{V}}_k(\widetilde{\mathbf{\Sigma}}_k)^{1/2};\\
&\mathbf{A}_1 = \mathbf{V}_{11}^*(\mathbf{\Sigma}_1^*)^{1/2};\ \mathbf{A}_2 = \mathbf{V}_{12}^*(\mathbf{\Sigma}_1^*)^{1/2};\ \mathbf{B}_1 = \mathbf{V}_{21}^*(\mathbf{\Sigma}_2^*)^{1/2};\ \mathbf{B}_2 = \mathbf{V}_{22}^*(\mathbf{\Sigma}_1^*)^{1/2};\\
&\widetilde{\mathbf{A}}_1 = \widetilde{\mathbf{V}}_{11}(\widetilde{\mathbf{\Sigma}}_1)^{1/2};\ \widetilde{\mathbf{A}}_2 = \widetilde{\mathbf{V}}_{12}(\widetilde{\mathbf{\Sigma}}_1)^{1/2};\ \widetilde{\mathbf{B}}_1 = \widetilde{\mathbf{V}}_{21}(\widetilde{\mathbf{\Sigma}}_2)^{1/2};\ \widetilde{\mathbf{B}}_2 = \widetilde{\mathbf{V}}_{22}(\widetilde{\mathbf{\Sigma}}_2)^{1/2}
\end{aligned} \tag{S.6}$$

and $\mathbf{Q}_A = \mathbf{G}(\widetilde{\mathbf{A}}^\top\mathbf{A})$, $\mathbf{Q}_B = \mathbf{G}(\widetilde{\mathbf{B}}^\top\mathbf{B})$, $\widetilde{\mathbf{O}} = \mathbf{G}(\widetilde{\mathbf{A}}_2^\top\widetilde{\mathbf{B}}_1)$. It is easy to see that

$$\widetilde{\mathbf{W}}_{sk} = \widetilde{\mathbf{A}}_1\widetilde{\mathbf{O}}^\top\widetilde{\mathbf{B}}_2^\top = \widetilde{\mathbf{A}}_1\mathbf{Q}_A(\mathbf{Q}_A^\top\widetilde{\mathbf{O}}^\top\mathbf{Q}_B)\mathbf{Q}_B^\top\widetilde{\mathbf{B}}_2^\top = \widetilde{\mathbf{A}}_1\mathbf{Q}_A\mathbf{G}(\mathbf{Q}_B^\top\widetilde{\mathbf{B}}_1^\top\widetilde{\mathbf{A}}_2\mathbf{Q}_A)\mathbf{Q}_B^\top\widetilde{\mathbf{B}}_2^\top. \tag{S.7}$$

Then by Proposition 2, we have

$$\begin{aligned}
&\|\widetilde{\mathbf{W}}_{sk} - \mathbf{W}^*_{s\backslash k,k\backslash s}\| = \|(\widetilde{\mathbf{A}}_1\mathbf{Q}_A)(\mathbf{Q}_A^\top\widetilde{\mathbf{O}}^\top\mathbf{Q}_B)(\mathbf{Q}_B^\top\widetilde{\mathbf{B}}_2^\top) - \mathbf{A}_1\mathbf{O}^\top\mathbf{B}_2^\top\|\\
=&\|(\widetilde{\mathbf{A}}_1\mathbf{Q}_A)(\mathbf{Q}_A^\top\widetilde{\mathbf{O}}^\top\mathbf{Q}_B)(\mathbf{Q}_B^\top\widetilde{\mathbf{B}}_2^\top) - \mathbf{A}_1(\mathbf{Q}_A^\top\widetilde{\mathbf{O}}^\top\mathbf{Q}_B)(\mathbf{Q}_B^\top\widetilde{\mathbf{B}}_2^\top)\\
&+\mathbf{A}_1(\mathbf{Q}_A^\top\widetilde{\mathbf{O}}^\top\mathbf{Q}_B)(\mathbf{Q}_B^\top\widetilde{\mathbf{B}}_2^\top) - \mathbf{A}_1(\mathbf{Q}_A^\top\widetilde{\mathbf{O}}^\top\mathbf{Q}_B)\mathbf{B}_2^\top\\
&+\mathbf{A}_1(\mathbf{Q}_A^\top\widetilde{\mathbf{O}}^\top\mathbf{Q}_B)\mathbf{B}_2^\top - \mathbf{A}_1\mathbf{O}^\top\mathbf{B}_2^\top\|\\
\leq&\|\widetilde{\mathbf{B}}_2\|\|\widetilde{\mathbf{A}}_1\mathbf{Q}_A - \mathbf{A}_1\| + \|\widetilde{\mathbf{A}}_1\|\|\widetilde{\mathbf{B}}_2\mathbf{Q}_B - \mathbf{B}_2\| + \|\mathbf{A}_1\|\|\mathbf{B}_2\|\|\mathbf{Q}_A^\top\widetilde{\mathbf{O}}^\top\mathbf{Q}_B - \mathbf{O}\|.
\end{aligned}$$

Applying Proposition S.9, Lemma S.6, Lemma S.7, Lemma S.10, with $f(p_0, N)$ defined in (S.18), we have

$$\begin{aligned}
&\|\widetilde{\mathbf{W}}_{sk} - \mathbf{W}^*_{s\backslash k,k\backslash s}\|\\
&\lesssim (1-p_0)(\|\mathbf{B}\|\|\widetilde{\mathbf{A}}\mathbf{Q}_A - \mathbf{A}\| + \|\mathbf{A}\|\|\widetilde{\mathbf{B}}\mathbf{Q}_B - \mathbf{B}\| + \|\mathbf{A}\|\|\mathbf{B}\|\|\mathbf{Q}_A^\top\widetilde{\mathbf{O}}^\top\mathbf{Q}_B - \mathbf{O}\|)\\
&\lesssim (1-p_0)\left\{r\mu_0\tau + f(p_0,N)^2(r\mu_0\tau)^2\right\}(\|\widetilde{\mathbf{E}}_1\| + \|\widetilde{\mathbf{E}}_2\|)\\
&\lesssim (1-p_0)(r\mu_0\tau)^2 f(p_0,N)^2\sqrt{Np_0}\sigma
\end{aligned}$$

with probability $1 - O(1/N^3)$.

S.2.3 Completion Error

After we impute the missing blocks, we can bound $\|\widehat{\mathbf{W}} - \mathbf{W}^*\|$ where $\widehat{\mathbf{W}}$ is defined as (9). Notice that

$$\widehat{\mathbf{W}} = \mathbf{W}^* + \widetilde{\mathbf{E}} + \widetilde{\mathbf{F}}, \tag{S.8}$$

where

$$\widetilde{\mathbf{E}} = \begin{bmatrix} \mathbf{E}^s_{s\setminus k, s\setminus k} & \mathbf{E}^s_{s\setminus k, s\cap k} & \mathbf{O} \\ \mathbf{E}^s_{s\cap k, s\setminus k} & \alpha_s \mathbf{E}^s_{s\cap k, s\cap k} + \alpha_k \mathbf{E}^k_{s\cap k, s\cap k} & \mathbf{E}^k_{s\cap k, k\setminus s} \\ \mathbf{O} & \mathbf{E}^k_{k\setminus s, s\cap k} & \mathbf{E}^k_{k\setminus s, k\setminus s} \end{bmatrix},$$

and

$$\widetilde{\mathbf{F}} = \begin{bmatrix} \mathbf{O} & \mathbf{O} & \widetilde{\mathbf{W}}_{sk} - \mathbf{W}^*_{s\setminus k, k\setminus s} \\ \mathbf{O} & \mathbf{O} & \mathbf{O} \\ \widetilde{\mathbf{W}}^\top_{sk} - \mathbf{W}^*_{k\setminus s, s\setminus k} & \mathbf{O} & \mathbf{O} \end{bmatrix}.$$

Then we only need to bound $\|\widetilde{\mathbf{E}}\|$ and $\|\widetilde{\mathbf{F}}\|$. It is easy to see that $\|\widetilde{\mathbf{F}}\| = \|\widetilde{\mathbf{W}}_{sk} - \mathbf{W}^*_{s\setminus k, k\setminus s}\|$, then we only need to bound $\|\widetilde{\mathbf{E}}\|$. It is easy to see that $\|\widetilde{\mathbf{E}}\| \le \|\mathbf{E}^s\| + \|\mathbf{E}^k\| \lesssim \sqrt{Np_0}\sigma$. However, to give some intuition on the choice of α_s and α_k, we consider a special case that the entries of $\mathbf{E}^s$ and $\mathbf{E}^k$ are independent mean zero sub-Gaussian random variables with sub-Gaussian norm σ. Then by Corollary 3.3 of Bandeira and van Handel (2016), we have

$$\mathbb{E}\|\widetilde{\mathbf{E}}\| \lesssim \sigma^* + \sigma\sqrt{\log N_0},$$

where $\sigma = \max\{\sigma_s, \sigma_k\}$ and $\sigma^* = \max_i \sqrt{\sum_j \mathbb{E}\widetilde{\mathbf{E}}^2_{ij}}$. It is easy to see that

$$\sigma^* = \max\{\sqrt{N_s}\sigma_s, \sqrt{N_k}\sigma_k, \sqrt{(N_s - N_{sk})\sigma_s^2 + (N_k - N_{sk})\sigma_k^2 + N_{sk}(\alpha_s^2\sigma_s^2 + \alpha_k^2\sigma_k^2)}\}.$$

In addition, by Lemma 11 and Proposition 1 of Chen and Wainwright (2015), there exists a universal constant $c > 0$ such that

$$\mathbb{P}\{\|\widetilde{\mathbf{E}}\| \ge c(\sigma^* + \sigma \log N_0)\} \le N_0^{-12}.$$

In order to minimize $\|\widetilde{\mathbf{E}}\|$ with regard to α_s and α_k, the best we can do is to minimize its upper bound. It is easy to see that

$$(\alpha_1^*, \alpha_2^*) = (\sigma_2^2/(\sigma_1^2 + \sigma_2^2), \sigma_1^2/(\sigma_1^2 + \sigma_2^2)) = \arg\min_{\alpha_1+\alpha_2=1, \alpha_1>0, \alpha_2>0} \alpha_1^2\sigma_1^2 + \alpha_2^2\sigma_2^2.$$

In reality, we do not know σ_s and σ_k, but we can estimate them by (10). Since

$$\alpha_1^2\sigma_1^2 + \alpha_2^2\sigma_2^2 \le (\alpha_1^2 + \alpha_2^2)\sigma^2 \le (\alpha_1 + \alpha_2)^2\sigma^2 = \sigma^2,$$

we have $\sigma^* \le \sqrt{N_0}\sigma$. So $\|\widetilde{\mathbf{E}}\| \lesssim \sigma^* \le \sqrt{N_0}\sigma$ with probability at least $1 - N_0^{-12} \ge 1 - O(1/N^3)$. By $N_0 = N_s + N_k - N_{sk} \le 3Np_s/2 + 3Np_k/2 - Np_sp_k/2 \lesssim Np_0$, we get $\sigma^* \lesssim \sqrt{Np_0}\sigma$. Finally, we have

$$\|\widehat{\mathbf{W}} - \mathbf{W}^*\| \le \|\widetilde{\mathbf{E}}\| + \|\widetilde{\mathbf{F}}\| \lesssim \sqrt{Np_0}\sigma + (1 - p_0)(r\mu_0\tau)^2 f(p_0, N)^2\sqrt{Np_0}\sigma.$$

The inequality still holds if the sub-Gaussian assumption does not hold.

S.2.4 Low-rank Approximation

The last step is to do rank-r eigendecomposition on $\widehat{\mathbf{W}}$ to obtain $\widehat{\mathbf{W}}_r = \widehat{\mathbf{U}}\widehat{\mathbf{\Sigma}}\widehat{\mathbf{U}}^\top = \widehat{\mathbf{X}}\widehat{\mathbf{X}}^\top$ where $\widehat{\mathbf{X}} = \widehat{\mathbf{U}}\widehat{\mathbf{\Sigma}}^{1/2}$. Then there exists an orthogonal matrix $\mathbf{O}_X$ such that

$$\begin{aligned}\|\widehat{\mathbf{X}}\mathbf{O}_X - \mathbf{X}^*\| &\lesssim \frac{\|\widehat{\mathbf{W}} - \mathbf{W}^*\| r\mu_0\tau}{\sqrt{\lambda_r(\mathbf{W}_0^*)}} \lesssim \frac{\|\widehat{\mathbf{W}} - \mathbf{W}^*\| r\mu_0\tau}{\sqrt{\lambda_{\min} p_0}} \\ &\lesssim \{(1-p_0)(r\mu_0\tau)^2 f(p_0, N)^2 + 1\} r\mu_0\tau\sqrt{\frac{N}{\lambda_{\min}}}\sigma.\end{aligned}$$

by a similar proof as Lemma S.7 and the fact that $\lambda_r(\mathbf{W}_0^*) \geq \lambda_r(\mathbf{W}_s^*) \geq p_0\lambda_{\min}/4$. Finally, this upper bound holds with probability at least $1 - O(1/N^3)$ by the probability union bound.

S.3. Proof of Theorem 12

We know that $N_0 \sim \text{Binomial}(N, 1 - \prod_{s=1}^m (1-p_s))$, so by the same argument to Lemma S.1, we have

$$N\{1 - \prod_{s=1}^m (1-p_s)\}/2 \leq N_0 \leq 3N\{1 - \prod_{s=1}^m (1-p_s)\}/2$$

with probability $1 - O(1/N^3)$. As a result,

$$\{1 - (1-p_0)^m\}\lambda_{\min} \lesssim \lambda_r(\mathbf{W}_0^*) \leq \lambda_1(\mathbf{W}_0^*) \lesssim \{1 - (1-p_0)^m\} r\mu_0\lambda_{\max} \qquad \text{(S.9)}$$

by a similar argument as in the proof of Theorem 7 and the Assumption 2 that $p_s/p_0 = O(1)$. In addition, let $\mathbf{E} = \widehat{\mathbf{W}} - \mathbf{W}_0^*$, then by a similar decomposition as in (S.8), we will have

$$\|\mathbf{E}\| \leq \|\widetilde{\mathbf{E}}\| + \sum_{s=1}^{m-1}\sum_{k=s+1}^{m} \|\mathbf{T}^{sk} \circ (\widetilde{\mathbf{W}}_{sk} - \mathbf{W}^*_{s\backslash k, k\backslash s})\|$$

where $\widetilde{\mathbf{E}} \in R^{n\times n}$ with

$$\widetilde{\mathbf{E}}(i,j) = \sum_{s=1}^m \alpha_{ij}^s \mathbf{E}^s(v_i^s, v_j^s)\mathbb{1}(i,j \in \mathcal{V}_s), \text{ for } \mathcal{S}_{ij} > 0$$

and $\widetilde{\mathbf{E}}(i,j) = 0$ for $\mathcal{S}_{ij} = 0$. Here we denote $\circ$ as the Hadamard product operator and $\mathbf{T}^{sk}, s \neq k \in [m]$ are 0/1 matrices decided by the Algorithm 1. According to the Algorithm 1, the nonzero entries of $\mathbf{T}^{sk}, s \neq k \in [m]$ are block-wise, which implies that

$$\|\mathbf{T}^{sk} \circ (\widetilde{\mathbf{W}}_{sk} - \mathbf{W}^*_{s\backslash k, k\backslash s})\| \leq \|\widetilde{\mathbf{W}}_{sk} - \mathbf{W}^*_{s\backslash k, k\backslash s}\|.$$

Then, by the proof of Theorem 7, we have $\|\widetilde{\mathbf{E}}\| \lesssim \sqrt{Np_0}\sigma$ and

$$\|\widetilde{\mathbf{W}}_{sk} - \mathbf{W}^*_{s\backslash k, k\backslash s}\| \lesssim (1-p_0)(r\mu_0\tau)^2 f(p_0, N)^2\sqrt{Np_0}\sigma$$

hold simultaneously with probability $1 - O(m^2/N^3)$ for $1 \leq s < k \leq m$. As a result,

$$\|\widehat{\mathbf{W}} - \mathbf{W}_0^*\| \lesssim m(m-1)(1-p_0)(r\mu_0\tau)^2 f(p_0, N)^2\sqrt{Np_0}\sigma + \sqrt{Np_0}\sigma$$

and

$$\|\widehat{\mathbf{X}}\mathbf{O}_X - \mathbf{X}^*\| \lesssim \frac{\|\widehat{\mathbf{W}} - \mathbf{W}_0^*\| r\mu_0\tau}{\sqrt{\lambda_r(\mathbf{W}_0^*)}}.$$

By (S.9), we have

$$\|\widehat{\mathbf{X}}\mathbf{O}_X - \mathbf{X}^*\| \lesssim \Big\{1 + m^2(1-p_0)(r\mu_0\tau)^2 f(p_0,N)^2\sqrt{\frac{p_0}{1-(1-p_0)^m}}\Big\} r\mu_0\tau\sqrt{\frac{N}{\lambda_{\min}}}\sigma$$

with probability $1 - O(m^2/N^3)$. Given $0 < \epsilon < 1$, we have

$$\mathbb{P}\{N_0 < (1-\epsilon)N\} = O\big(\frac{1}{N^3}\big)$$

when $m \approx \log(\epsilon - \sqrt{\frac{3\log N}{2N}})/\log(1-p_0)$ by the fact that $N_0 \sim \text{Binomial}(N, 1-\prod_{s=1}^m(1-p_s))$ and the Bernstein inequality. Since $\lim_{N\to\infty}\sqrt{\log N/N} = 0$ we have $m \approx \log\epsilon/\log(1-p_0)$. Finally, we have

$$\|\widehat{\mathbf{X}}\mathbf{O}_X - \mathbf{X}^*\| \lesssim \Big\{1 + \frac{\log^2\epsilon}{\log^2(1-p_0)}(1-p_0)(r\mu_0\tau)^2 f(p_0,N)^2\sqrt{\frac{p_0}{1-(1-p_0)^m}}\Big\} r\mu_0\tau\sqrt{\frac{N}{\lambda_{\min}}}\sigma$$

with probability $1 - O(m^2/N^3)$.

S.4. Details of the Proof of Theorem 7

Here we present some key lemmas and propositions needed for our proof of Theorem 7.

Lemma S.1 (The dimension of the sub-matrices) *Under the assumption that*

$$p_s \geq p_0 \geq C\sqrt{\mu_0 r\tau\log N/N},$$

for some sufficiently large constant C, we have

$$\frac{p_sN}{2} \leq N_s \leq \frac{3p_sN}{2} \text{ and } \frac{p_sp_kN}{2} \leq N_{sk} \leq \frac{3p_sp_kN}{2}, s \neq k, s, k \in [m] \tag{S.10}$$

with probabilities $1 - O(m^2/N^3)$.

Proof By the Bernstein inequality, we have

$$\mathbb{P}\{Y \leq pn - t\} \leq \exp\{-\frac{\frac{1}{2}t^2}{np(1-p)+\frac{1}{3}t}\} \text{ and } \mathbb{P}\{Y \geq pn + t\} \leq \exp\{-\frac{\frac{1}{2}t^2}{np(1-p)+\frac{1}{3}t}\}$$

if $Y \sim \text{Binomial}(n,p)$. Since $N_s \sim \text{Binomial}(N, p_s)$ and $N_{sk} \sim \text{Binomial}(N, p_sp_k)$, let $t = \frac{p_s}{2}$, we have

$$\mathbb{P}\{\frac{p_sN}{2} \leq N_s \leq \frac{3p_sN}{2}\} \geq 1 - 2\exp\{-\frac{3p_sN}{28}\}.$$

Similarly, we have

$$\mathbb{P}\{\frac{p_sp_kN}{2} \leq N_{sk} \leq \frac{3p_sp_kN}{2}\} \geq 1 - 2\exp\{-\frac{3p_sp_kN}{28}\}.$$

In addition, by $p_s \geq p_0 \geq C\sqrt{\mu_0 r\tau \log N/N}$, we have $\exp\{-3p_sN/28\} = O(1/N^3)$ and $\exp\{-3p_sp_kN/28\} = O(1/N^3)$. Finally, by the probability union bound, (S.10) holds with probability $1 - O(m^2/N^3)$. ■

Lemma S.2 (Lemma 5, Cai et al. (2016)) *Suppose $\mathbf{U} \in \mathbb{R}^{N\times r}$ ($N \geq r$) is a fixed matrix with orthonormal columns. Denote $\mu = \max_{1\leq i\leq N} \frac{N}{r}\sum_{j=1}^r u_{ij}^2$. Suppose we uniform randomly draw n rows (with or without replacement) from $\mathbf{U}$ and denote it as $\mathbf{U}_\Omega$, where Ω is the index set. When $n \geq 4\mu r(\log r + c)/(1-\alpha)^2$ for some $0 < \alpha < 1$ and $c > 1$, we have*

$$\sigma_{\min}(\mathbf{U}_\Omega) \geq \sqrt{\frac{\alpha n}{N}}$$

with probability $1 - 2e^{-c}$.

By Lemma S.2, we will directly have the following proposition.

Proposition S.3 *Let $\alpha = \frac{1}{2}$ and $c = \log 2N^3$ in Lemma S.2, then when*

$$N_s \geq 16\mu_0 r(\log r + \log 2N^3),$$

we have $\sigma_{\min}(\mathbf{U}^*_{\mathcal{V}_s}) \geq \sqrt{\frac{N_s}{2N}}$ with probability $1-1/N^3$. In addition, under the event, we have

$$\lambda_r(\mathbf{W}_s^*) = \lambda_r(\mathbf{U}^*_{\mathcal{V}_s}\mathbf{\Sigma}^*(\mathbf{U}^*_{\mathcal{V}_s})^\top) \geq \sigma_{\min}(\mathbf{U}^*_{\mathcal{V}_s})\lambda_r(\mathbf{\Sigma}^*)\sigma_{\min}(\mathbf{U}^*_{\mathcal{V}_s}) \geq \frac{N_s\lambda_{\min}}{2N}.$$

Lemma S.4 (Incoherence condition of the sub-matrices) *Recall that $\mathbf{V}_s^*\mathbf{\Sigma}_s^*(\mathbf{V}_s^*)^\top$ is the rank-r eigendecomposition of $\mathbf{W}_s^*$. Assume that $\lambda_r(\mathbf{W}_s^*) \geq \frac{N_s\lambda_{\min}}{2N}$. Then the incoherence of $\mathbf{V}_s^*$ satisfies*

$$\mu_s \equiv \mu(\mathbf{V}_s^*) = \frac{N_s}{r}\max_{i=1,\ldots,n_s}\sum_{j=1}^r \mathbf{V}_s^*(i,j)^2 \leq 2\tau\mu_0.$$

Proof Since $\mathbf{W}_s^* = \mathbf{U}^*_{\mathcal{V}_s}\mathbf{\Sigma}^*(\mathbf{U}^*_{\mathcal{V}_s})^\top = \mathbf{V}_s^*\mathbf{\Sigma}_s^*(\mathbf{V}_s^*)^\top$, we have

$$\mathbf{V}_s^* = \mathbf{U}^*_{\mathcal{V}_s}(\mathbf{\Sigma}^*)^{\frac{1}{2}}\mathbf{O}_s^\top(\mathbf{\Sigma}_s^*)^{-\frac{1}{2}}$$

where $\mathbf{O}_s = (\mathbf{\Sigma}_s^*)^{-\frac{1}{2}}(\mathbf{V}_s^*)^\top\mathbf{U}^*_{\mathcal{V}_s}(\mathbf{\Sigma}^*)^{\frac{1}{2}} \in \mathscr{O}^{r\times r}$. Then

$$\sum_{j=1}^r \mathbf{V}_s^*(i,j)^2 \leq \sum_{j=1}^r \mathbf{U}^*_{\mathcal{V}_s}(i,j)^2\|(\mathbf{\Sigma}_s^*)^{-\frac{1}{2}}\|^2\|(\mathbf{\Sigma}^*)^{\frac{1}{2}}\|^2 \leq \frac{r\mu_0}{N}\frac{\lambda_{\max}}{\lambda_r(\mathbf{W}_s^*)}$$

As a result,

$$\mu_s = \frac{N_s}{r}\max_{i=1,\ldots,n_s}\sum_{j=1}^r \mathbf{V}_s^*(i,j)^2 \leq \frac{N_s\mu_0}{N}\frac{\sigma_{\max}}{\sigma_r(\mathbf{W}_s^*)} \leq \frac{2\lambda_{\max}}{\lambda_{\min}}\mu_0 = 2\tau\mu_0.$$

■

Proposition S.5 (Upper bound of the operator norm of the sub-matrices) *We have*

$$\lambda_1(\mathbf{W}_s^*) \le \min\{1, \frac{N_s r \mu_0}{N}\}\lambda_{\max} \text{ for } s \in [m].$$

Proof It is obviously that $\lambda_1(\mathbf{W}_s^*) = \lambda_1(\mathbf{U}_{\mathcal{V}_s}^* \mathbf{\Sigma}^* (\mathbf{U}_{\mathcal{V}_s}^*)^\top) \le \sigma_{\max}(\mathbf{U}_{\mathcal{V}_s}^*)^2 \lambda_{\max}(\mathbf{\Sigma}^*) \le \lambda_{\max}$ because $\sigma_{\max}(\mathbf{U}_{\mathcal{V}_s}^*) \le 1$. Besides, we have $\|\mathbf{U}_{\mathcal{V}_s}^*\|^2 \le N_s \|\mathbf{U}_{\mathcal{V}_s}^*\|_{2,\infty}^2 \le N_s r \mu_0 / N$ where the first inequality comes from the property of ℓ_2/ℓ_∞ norm and the second inequality comes from $\mu_0 = \mu(\mathbf{U}^*)$ and the definition of incoherence. ∎

S.4.1 Error Matrix

Recalling that $\widetilde{\mathbf{W}}_s \equiv \widetilde{\mathbf{W}}_{\mathcal{V}_s,\mathcal{V}_s}$, we characterize the operator norm of $\widetilde{\mathbf{W}}_s - \mathbf{W}_s^*, s \in [m]$ in the Lemma S.6.

Lemma S.6 *Let $\widetilde{\mathbf{E}}_s := \widetilde{\mathbf{W}}_s - \mathbf{W}_s^*, s \in [m]$. Under Assumptions 2, 3, and the condition $p_s N/2 \le n_s \le 3p_s N/2, s \in [m]$, we have*

$$\|\widetilde{\mathbf{E}}_s\| \lesssim \sqrt{Np_0}\sigma \ll \frac{p_0 \lambda_{\min}}{4} \le \lambda_r(\mathbf{W}_s^*), s \in [m]$$

with probability $1 - O(m/N^3)$.

Proof Recall that

$$\widetilde{\mathbf{W}}_s(v_i^s, v_j^s) = \widetilde{\mathbf{W}}(i,j) = \sum_{k=1}^m \alpha_{ij}^k \mathbf{W}^k(v_i^k, v_j^k) \mathbb{1}(i, j \in \mathcal{V}_k), i, j \in \mathcal{V}_s.$$

We then have

$$\widetilde{\mathbf{E}}_s(v_i^s, v_j^s) = \sum_{k=1}^m \alpha_{ij}^k \mathbf{E}^k(v_i^k, v_j^k) \mathbb{1}(i, j \in \mathcal{V}_s), i, j \in \mathcal{V}_s$$

and it is easy to see $\|\widetilde{\mathbf{E}}_s\| \le \max_{k\in[m]} \|\widehat{\mathbf{E}}^k\| \lesssim \sqrt{N_s}\sigma, s \in [m]$. In addition, $N_s \le 3p_s N/2$ leads to

$$\|\widetilde{\mathbf{E}}_s\| \lesssim \sqrt{Np_0}\sigma, s \in [m]$$

with probability at least $1 - O(m/N^3)$, and based on Assumption 3, we have

$$\|\widetilde{\mathbf{E}}_s\| \ll \frac{p_0 \lambda_{\min}}{4} \le \lambda_r(\mathbf{W}_s^*),\ s \in [m].$$

∎

We then bound $\|\widetilde{\mathbf{A}}\mathbf{Q}_A - \mathbf{A}\|$ and $\|\widetilde{\mathbf{B}}\mathbf{Q}_B - \mathbf{B}\|$ for the case $m = 2$ in the following lemma.

Lemma S.7 *Based on the notation on Section S.2.2 with the assumptions that $\|\widetilde{\mathbf{E}}_l\| \ll \lambda_r(\mathbf{W}_l^*)$ and $\tau_l = \lambda_1(\mathbf{W}_l^*)/\lambda_r(\mathbf{W}_l^*), l = s, k$ are bounded, we have*

$$\|\widetilde{\mathbf{A}}\mathbf{Q}_A - \mathbf{A}\| \lesssim \frac{\tau_s}{\sqrt{\lambda_r(\mathbf{W}_s^*)}} \|\widetilde{\mathbf{E}}_s\| \quad \text{and} \quad \|\widetilde{\mathbf{B}}\mathbf{Q}_B - \mathbf{B}\| \lesssim \frac{\tau_k}{\sqrt{\lambda_r(\mathbf{W}_k^*)}} \|\widetilde{\mathbf{E}}_k\|.$$

Proof Define $\mathbf{Q}_s = \mathbf{G}(\widetilde{\mathbf{V}}_s^\top \mathbf{V}_s^*)$, $\mathbf{Q}_k = \mathbf{G}(\widetilde{\mathbf{V}}_k^\top \mathbf{V}_k^*)$ and recall that $\mathbf{Q}_A = \mathbf{G}(\widetilde{\mathbf{A}}^\top \mathbf{A})$ and $\mathbf{Q}_B = \mathbf{G}(\widetilde{\mathbf{B}}^\top \mathbf{B})$. The key decomposition we need is the following:

$$\widetilde{\mathbf{A}}\mathbf{Q}_A - \mathbf{A} = \widetilde{\mathbf{A}}(\mathbf{Q}_A - \mathbf{Q}_s) + \widetilde{\mathbf{V}}_s[\widetilde{\mathbf{\Sigma}}_s^{\frac{1}{2}}\mathbf{Q}_s - \mathbf{Q}_s(\mathbf{\Sigma}_s^*)^{\frac{1}{2}}] + (\widetilde{\mathbf{V}}_s\mathbf{Q}_s - \mathbf{V}_s^*)(\mathbf{\Sigma}_s^*)^{\frac{1}{2}}. \tag{S.11}$$

For the spectral norm error bound, the triangle inequality together with (S.11) yields

$$\|\widetilde{\mathbf{A}}\mathbf{Q}_A - \mathbf{A}\| \le \|\widetilde{\mathbf{\Sigma}}_s^{\frac{1}{2}}\|\|\mathbf{Q}_A - \mathbf{Q}_s\| + \|\widetilde{\mathbf{\Sigma}}_s^{\frac{1}{2}}\mathbf{Q}_s - \mathbf{Q}_s(\mathbf{\Sigma}_s^*)^{\frac{1}{2}}\| + \sqrt{\lambda_1(\mathbf{\Sigma}_s^*)}\|\widetilde{\mathbf{V}}_s\mathbf{Q}_s - \mathbf{V}_s^*\|,$$

where we have also used the fact that $\|\widetilde{\mathbf{V}}_s\| = 1$. Recognizing that $\|\widetilde{\mathbf{W}}_s - \mathbf{W}_s^*\| = \|\widetilde{\mathbf{E}}_s\| \ll \lambda_r(\mathbf{W}_s^*)$ and the assumption that $\lambda_1(\mathbf{W}_s^*)/\lambda_r(\mathbf{W}_s^*)$ is bounded, we can apply Lemmas 47, 46, 45 of Ma et al. (2018) to obtain

$$\|\mathbf{Q}_A - \mathbf{Q}_s\| \lesssim \frac{1}{\lambda_r(\mathbf{W}_s^*)}\|\widetilde{\mathbf{E}}_s\|,$$

$$\|\widetilde{\mathbf{\Sigma}}_s^{\frac{1}{2}}\mathbf{Q}_s - \mathbf{Q}_s(\mathbf{\Sigma}_s^*)^{\frac{1}{2}}\| \lesssim \frac{1}{\sqrt{\lambda_r(\mathbf{W}_s^*)}}\|\widetilde{\mathbf{E}}_s\|,$$

$$\|\widetilde{\mathbf{V}}_s\mathbf{Q}_s - \mathbf{V}_s^*\| \lesssim \frac{1}{\lambda_r(\mathbf{W}_s^*)}\|\widetilde{\mathbf{E}}_s\|.$$

These taken collectively imply the advertised upper bound

$$\|\widetilde{\mathbf{A}}\mathbf{Q}_A - \mathbf{A}\| \lesssim \frac{\sqrt{\lambda_1(\mathbf{W}_s^*)}}{\lambda_r(\mathbf{W}_s^*)}\|\widetilde{\mathbf{E}}_s\| + \frac{1}{\sqrt{\lambda_r(\mathbf{W}_s^*)}}\|\widetilde{\mathbf{E}}_s\| \lesssim \frac{\sqrt{\tau_s}}{\sqrt{\lambda_r(\mathbf{W}_s^*)}}\|\widetilde{\mathbf{E}}_s\|,$$

where we also utilize the fact that $\|\widetilde{\mathbf{\Sigma}}_s\| \le \|\mathbf{\Sigma}_s^*\| + \|\widetilde{\mathbf{E}}_s\| \le 2\|\mathbf{\Sigma}_s^*\| = 2\|\mathbf{W}_s^*\|$ and $\lambda_1(\mathbf{W}_s^*)/\lambda_r(\mathbf{W}_s^*)$ is bounded. Similarly, we have

$$\|\widetilde{\mathbf{B}}\mathbf{Q}_B - \mathbf{B}\| \lesssim \frac{\sqrt{\tau_k}}{\sqrt{\lambda_r(\mathbf{W}_k^*)}}\|\widetilde{\mathbf{E}}_k\|.$$

Combined with the fact that $\tau_l = \lambda_1(\mathbf{W}_l^*)/\lambda_r(\mathbf{W}_l^*) \le 6r\mu_0\tau, l = s, k$, we have

$$\|\widetilde{\mathbf{A}}\mathbf{Q}_A - \mathbf{A}\| \lesssim \frac{\sqrt{r\mu_0\tau}}{\sqrt{\lambda_r(\mathbf{W}_s^*)}}\|\widetilde{\mathbf{E}}_s\| \quad \text{and} \quad \|\widetilde{\mathbf{B}}\mathbf{Q}_B - \mathbf{B}\| \lesssim \frac{\sqrt{r\mu_0\tau}}{\sqrt{\lambda_r(\mathbf{W}_k^*)}}\|\widetilde{\mathbf{E}}_k\|.$$

■

S.4.2 Probability Bound for Submatrix

Lemma S.8 *Denote $\mathbf{R} \in \mathbb{R}^{d\times d}$ for the square diagonal matrix whose jth diagonal entry is y_j, where $\{y_j\}_{j=1}^n$ is a sequence of independent $0-1$ random variables with common mean p. Let $\mathbf{B} \in \mathbb{R}^{q\times d}$ with rank r and $d > \max\{e^2, r^2\}$.*

- *If $p = o(1/\log d)$ or p is bounded away from 0 for all d, we have*

$$\mathbb{P}\{\|\mathbf{BR}\| \ge Cp^{\frac{1}{2}}\|\mathbf{B}\|\} \le \delta \tag{S.12}$$

- *else,*

$$\mathbb{P}\{\|\mathbf{BR}\| \geq Cp^{\frac{1}{2}}\sqrt{p\log d}\|\mathbf{B}\|\} \leq \delta \tag{S.13}$$

for some universal positive constant C *and* $\delta = 1/d^3$.

Proof By Theorems 3.1 and 4.1 of Tropp (2008), we have

$$\mathbb{E}_k\|\mathbf{BR}\| \leq 6\sqrt{\max\{k, 2\log r\}}\frac{p}{1-p}\max_{|T|\leq p^{-1}}[\sum_{j\in T}\|\boldsymbol{b}_j\|_2^k]^{1/k} + \sqrt{p}\|\mathbf{B}\|. \tag{S.14}$$

for $k \in [2,\infty)$ where $\mathbb{E}_k\mathbf{X} = (\mathbb{E}|\mathbf{X}|^k)^{1/k}$ and the ℓ_1 to ℓ_2 operator norm $\|\cdot\|_{1\to2}$ computes the maximum ℓ_2 norm of a column. In addition, $\boldsymbol{b}_j$ is the jth column of $\mathbf{B}$ and $T \subset [d]$. Since $\|\boldsymbol{b}_j\|_2 \leq \|\mathbf{B}\|$, we have

$$\max_{|T|\leq p^{-1}}[\sum_{j\in T}\|\boldsymbol{b}_j\|_2^k]^{1/k} \leq (p^{-1}\|\mathbf{B}\|^k)^{1/k} = p^{-1/k}\|\mathbf{B}\|.$$

As a result,

$$\mathbb{E}_k\|\mathbf{BR}\| \leq p^{\frac{1}{2}}\{\frac{6\sqrt{\max\{k, 2\log r\}}p^{\frac{1}{2}-\frac{1}{k}}}{1-p} + 1\}\|\mathbf{B}\| \tag{S.15}$$

for $k \in [2,\infty)$. In addition, it is obviously that $\mathbb{E}_k\|\mathbf{BR}\| \leq \|\mathbf{B}\|$. When $p \geq \frac{1}{2}$, we have

$$p^{\frac{1}{2}}\{\frac{6\sqrt{\max\{k, 2\log r\}}p^{\frac{1}{2}-\frac{1}{k}}}{1-p} + 1\} \geq p^{\frac{1}{2}}\{12\sqrt{2\log r}p^{\frac{1}{2}-\frac{1}{k}} + 1\} \geq \frac{1}{\sqrt{2}}\{12\sqrt{\log r} + 1\} > 1$$

and when $p < \frac{1}{2}$ we have

$$p^{\frac{1}{2}}\{\frac{6\sqrt{\max\{k, 2\log r\}}p^{\frac{1}{2}-\frac{1}{k}}}{1-p} + 1\} < p^{\frac{1}{2}}\{12\max\sqrt{\{k, 2\log r\}}p^{\frac{1}{2}-\frac{1}{k}} + 1\}.$$

As a result, we have

$$\mathbb{E}_k\|\mathbf{BR}\| \leq c_1(p, r, k)\|\mathbf{B}\|$$

where $c_1(p,r,k) = \min\{1, p^{\frac{1}{2}}\{12\sqrt{\max\{k, 2\log r\}}p^{\frac{1}{2}-\frac{1}{k}}+1\}\}$. Let $k_0 = \log d \geq 2\log r$. Then by Markov inequality, we have

$$\mathbb{P}\{\|\mathbf{BR}\| \geq p^{\frac{1}{2}}\{\delta^{-1/k_0}c_1(p, r, k_0)/\sqrt{p}\}\|\mathbf{B}\|\} \leq \delta. \tag{S.16}$$

We discuss the (S.16) dependent on the conditions of p.

Case 1: $0 < p < c_3/\log d$ for all $d > 0$ and some fixed constant $c_3 > 0$. Then $\delta^{-1/q_0} = e^3$ is a constant. In addition, $\sqrt{k_0}p^{\frac{1}{2}-\frac{1}{k_0}} \leq \sqrt{c_3}\{c_3/\log d\}^{-1/\log d} < c_4$ for some constant c_4 since $\lim_{x\to\infty} x^{1/x} = 1$ is bounded. As a result, $c_1(p, r, k_0)/\sqrt{p} \leq 12c_4 + 1$ is also bounded.

Case 2: $p \geq c_5$ for all $d > 0$ and some fixed constant $0 < c_5 < 1$. Then let $c_6 = 1/\sqrt{c_5}$ and we have

$$\mathbb{P}\{\|\mathbf{BR}\| > p^{\frac{1}{2}}c_6\|\mathbf{B}\|\} \leq \delta \tag{S.17}$$

since $\|\mathbf{BR}\| \leq \|\mathbf{B}\|$ almost surely.

Case 3: $p = g(d)/\log d$ for some function $g(d) > 0$ which satisfies $\lim_{d\to\infty} g(d) = \infty$ and $\lim_{d\to\infty} g(d)/\log d = 0$. We still have $\delta^{-1/k_0} = e^3$. In addition, $c_1(p,r,k_0)/\sqrt{p} \le 12\sqrt{k_0}p^{\frac{1}{2}-\frac{1}{k_0}} + 1 \le 12\sqrt{g(d)}(\frac{\log d}{g(d)})^{1/\log d} + 1 \le c_7\sqrt{g(d)} = c_7\sqrt{p\log d}$ for some constant c_7 since $(\log d/g(d))^{1/\log d}$ is bounded.

Based on Case 1, 2 and 3, letting $C = \max\{e^3(12c_4+1), c_6, e^3c_7\}$, we will get the result. ■

Let $c_1 = \lim_{N\to\infty} p_0$ and $c_2 = \lim_{N\to\infty} p_0 \log N$. Define

$$f(p_0, N) = \mathbb{1}(c_1 > 0 \text{ or } c_2 = 0) + \{1 - \mathbb{1}(c_1 > 0 \text{ or } c_2 = 0)\}\sqrt{p_0 \log N}. \tag{S.18}$$

Then we have the following proposition.

Proposition S.9 *Based on the definition of* (S.7)*, under the assumption that* p_0 *is bounded away from 1, e.g.,* $\lim_{N_0\to\infty} p_0 < 1$*, directly apply Lemma S.8, we will get*

$$\begin{aligned}
\|\widetilde{\mathbf{A}}_1\mathbf{Q}_A - \mathbf{A}_1\| \lesssim \sqrt{1-p_0}\|\widetilde{\mathbf{A}}\mathbf{Q}_A - \mathbf{A}\|; &\quad \|\widetilde{\mathbf{A}}_2\mathbf{Q}_A - \mathbf{A}_2\| \lesssim \sqrt{p_0}f(p_0,N)\|\widetilde{\mathbf{A}}\mathbf{Q}_A - \mathbf{A}\|;\\
\|\widetilde{\mathbf{B}}_2\mathbf{Q}_B - \mathbf{B}_2\| \lesssim \sqrt{1-p_0}\|\widetilde{\mathbf{B}}\mathbf{Q}_B - \mathbf{B}\|; &\quad \|\widetilde{\mathbf{B}}_1\mathbf{Q}_A - \mathbf{B}_1\| \lesssim \sqrt{p_0}f(p_0,N)\|\widetilde{\mathbf{B}}\mathbf{Q}_B - \mathbf{B}\|;\\
\|\widetilde{\mathbf{A}}_1\| \lesssim \sqrt{1-p_0}\|\widetilde{\mathbf{A}}\|; &\quad \|\mathbf{A}_1\| \lesssim \sqrt{1-p_0}\|\mathbf{A}\|;\\
\|\mathbf{A}_2\| \lesssim \sqrt{p_0}f(p_0,N)\|\mathbf{A}\|; &\quad \|\widetilde{\mathbf{B}}_1\| \lesssim \sqrt{p_0}f(p_0,N)\|\widetilde{\mathbf{B}}\|;\\
\|\widetilde{\mathbf{B}}_2\| \lesssim \sqrt{1-p_0}\|\widetilde{\mathbf{B}}\|; &\quad \|\mathbf{B}_2\| \lesssim \sqrt{1-p_0}\|\mathbf{B}\|;
\end{aligned} \tag{S.19}$$

with probability $1 - 10/N^3$.

S.4.3 Orthogonal Procrustes Problem

Lemma S.10 (Orthogonal Procrustes problem) *Based on the definition of* (S.7)*, the condition of* (S.19)*, Assumption 2,* $\lambda_1(\mathbf{W}_l^*) \le 3p_0r\mu_0/2\lambda_{\max}$*,* $\lambda_r(\mathbf{W}_l^*) \ge p_l\lambda_{\min}/4$*,* $\|\widetilde{\mathbf{E}}_l\| \ll \lambda_r(\mathbf{W}_l^*), l = s,k,$ *and* $N_{sk} \ge 64r\mu_0\tau(\log r + \log 2N^3)$*, we have*

$$\|\mathbf{Q}_B^\top\widetilde{\mathbf{O}}\mathbf{Q}_A - \mathbf{O}\| \lesssim \frac{f(p_0,N)^2 r\mu_0\tau}{p_0\lambda_{\min}}\{\|\widetilde{\mathbf{E}}_s\| + \|\widetilde{\mathbf{E}}_k\|\} \tag{S.20}$$

with probability $1 - 2/N^3$.

Proof First,

$$\begin{aligned}
&\|\mathbf{A}_2^\top\mathbf{B}_1 - \mathbf{Q}_A^\top\widetilde{\mathbf{A}}_2^\top\widetilde{\mathbf{B}}_1\mathbf{Q}_B\| \le \|\mathbf{A}_2\|\|\widetilde{\mathbf{B}}_1\mathbf{Q}_B - \mathbf{B}_1\| + \|\widetilde{\mathbf{B}}_1\|\|\widetilde{\mathbf{A}}_2\mathbf{Q}_A - \mathbf{A}_2\|\\
&\le p_0f(p_0,N)^2\{\|\mathbf{A}\|\|\widetilde{\mathbf{B}}\mathbf{Q}_B - \mathbf{B}\| + \|\widetilde{\mathbf{B}}\|\|\widetilde{\mathbf{A}}\mathbf{Q}_A - \mathbf{A}\|\}\\
&\le 2p_0f(p_0,N)^2\{\|\mathbf{A}\|\|\widetilde{\mathbf{B}}\mathbf{Q}_B - \mathbf{B}\| + \|\mathbf{B}\|\|\widetilde{\mathbf{A}}\mathbf{Q}_A - \mathbf{A}\|\}\\
&\lesssim p_0f(p_0,N)^2\{\sqrt{\frac{r\mu_0\tau\lambda_1(\mathbf{W}_s^*)}{\lambda_r(\mathbf{W}_k^*)}}\|\widetilde{\mathbf{E}}_k\| + \sqrt{\frac{r\mu_0\tau\lambda_1(\mathbf{W}_k^*)}{\lambda_r(\mathbf{W}_s^*)}}\|\widetilde{\mathbf{E}}_s\|\}\\
&\le p_0f(p_0,N)^2r\mu_0\tau\{\|\widetilde{\mathbf{E}}_s\| + \|\widetilde{\mathbf{E}}_k\|\}
\end{aligned}$$

where the second inequality comes from (S.19), the third inequality comes from $\|\widetilde{\mathbf{B}}\| \leq \sqrt{\|\mathbf{W}_k^*\| + \|\widetilde{\mathbf{E}}_k\|} \leq \sqrt{2\|\mathbf{W}_k^*\|} \leq 2\|\mathbf{B}\|$ and the last inequality comes from Lemma S.7. In addition, since

$$\begin{aligned}\sigma_{r-1}(\mathbf{A}_2^\top \mathbf{B}_1) \geq \sigma_r(\mathbf{A}_2^\top \mathbf{B}_1) &= \sigma_r((\mathbf{V}_{s2}^*)^\top (\mathbf{\Sigma}_s^*)^{1/2} (\mathbf{\Sigma}_k^*)^{1/2} \mathbf{V}_{k1}^*) \\ &\geq \sigma_{\min}(\mathbf{V}_{s2}^*) \sqrt{\lambda_r(\mathbf{\Sigma}_s^*)\lambda_r(\mathbf{\Sigma}_k^*)} \sigma_{\min}(\mathbf{V}_{k1}^*)\end{aligned}$$

and again by $p_0 \geq C\sqrt{\mu_0 r\tau \log N/N}$, we will have $p_0 \geq \sqrt{64 r\mu_0\tau(\log r + \log 2N^3)/N}$. Then by Lemma S.2, $\sigma_{\min}(\mathbf{V}_{s2}^*)\sigma_{\min}(\mathbf{V}_{k1}^*) \geq p_0/6$ holds with probability $1 - 2/N^3$. Then

$$\sigma_{r-1}(\mathbf{A}_2^\top \mathbf{B}_1) \geq \sigma_r(\mathbf{A}_2^\top \mathbf{B}_1) \geq p_0^2 \lambda_{\min}/24.$$

So we can apply Lemma 23 of Ma et al. (2018) to get

$$\begin{aligned}\|\mathbf{Q}_A^\top \widetilde{\mathbf{O}} \mathbf{Q}_B - \mathbf{O}\| &\leq \frac{\|\mathbf{A}_2^\top \mathbf{B}_1 - \mathbf{Q}_A^\top \widetilde{\mathbf{A}}_2^\top \widetilde{\mathbf{B}}_1 \mathbf{Q}_B\|}{\sigma_{r-1}(\mathbf{A}_2^\top \mathbf{B}_1) + \sigma_r(\mathbf{A}_2^\top \mathbf{B}_1)} \\ &\leq \frac{p_0 f(p_0, N)^2 r\mu_0\tau}{2p_0^2\lambda_{\min}/24}\{\|\widetilde{\mathbf{E}}_s\| + \|\widetilde{\mathbf{E}}_k\|\} \lesssim \frac{f(p_0, N)^2 r\mu_0\tau}{p_0\lambda_{\min}}\{\|\widetilde{\mathbf{E}}_s\| + \|\widetilde{\mathbf{E}}_k\|\}.\end{aligned} \tag{S.21}$$

■

S.5. Discussion about Dependent Sampling

In this section, we explore the relaxation of the independence model (1) to accommodate a wider range of scenarios. To do this, we introduce a dependent model

$$\{p_s\}_{s=1}^m \sim P^m, \tag{S.22}$$

where the probabilities $\{p_s\}_{s=1}^m$ are drawn from an m-dimension distribution P^m over the sample space $(C\sqrt{\mu_0 r \log N/N}, 1)^m$ with a sufficiently large constant C. There are no other restrictions on the distribution P^m, so $\{p_s\}_{s=1}^m$ can be highly dependent. Conditioning on $\{p_s\}_{s=1}^m$, we assume that

$$\mathbb{I}(w \in \mathcal{V}_s \mid p_s) \text{ for } w \in \mathcal{V} \text{ and } s \in [m] \text{ independently, and } \mathbb{P}(w \in \mathcal{V}_s \mid p_s) = p_s. \tag{S.23}$$

This model allows the dependence for the emergence of corpora which is decided by P^m. Specifically, we have

$$\mathbb{P}(w_1 \in \mathcal{V}_s, w_2 \in \mathcal{V}_s) \neq \mathbb{P}(w_1 \in \mathcal{V}_s)\mathbb{P}(w_2 \in \mathcal{V}_s)$$

when $\mathrm{Var}(p_s) > 0$ and

$$\mathbb{P}(w_1 \in \mathcal{V}_s, w_2 \in \mathcal{V}_k) \neq \mathbb{P}(w_1 \in \mathcal{V}_s)\mathbb{P}(w_2 \in \mathcal{V}_k)$$

when $\mathrm{Cov}(p_s, p_k) \neq 0$ for $s \neq k$. As a result, if w_1 belongs to $\mathcal{V}_s$, it may influence the occurrence probability of other codes for $\mathcal{V}_s$ and $\mathcal{V}_k$ for $k \neq s$.

Despite these changes, our theorems still hold under the model defined by (S.22) and (S.23). Specifically, conditioning on $\{p_s\}_{s=1}^m$, we can still guarantee the overlapping matrices are of rank r and apply Lemma S.8. Following the same analysis, we can prove the same bounds as Theorems 7 and 12.

S.6. Additional Simulation Results

To validate the data-driven method for choosing r in Section 5, we rerun all simulations in Section 4 while only replacing the true rank with the estimated one for all methods. We observe a similar pattern to the results in Section 4.3, and BONMI still performs the best. The results are presented in Figures 4.

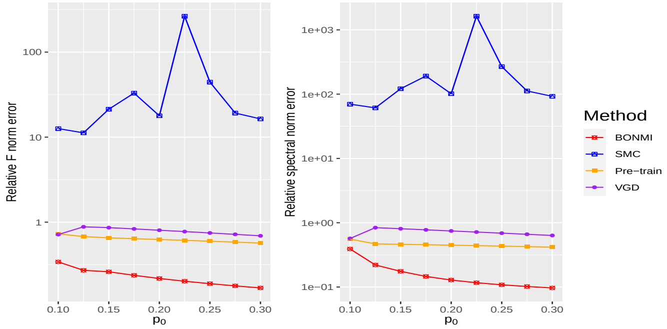

(a) setting (i): fix $m = 2$ and range p_0 from 0.1 to 0.3.

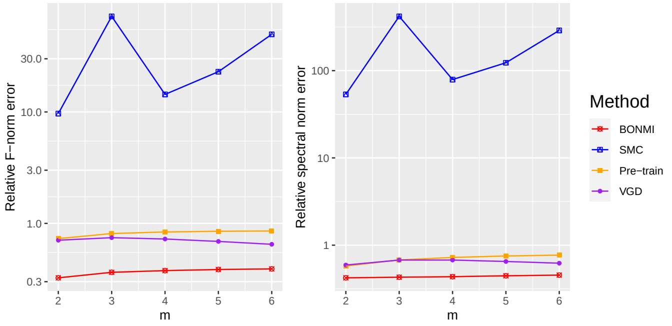

(b) setting (ii): fix $p_0 = 0.1$ and range m from 2 to 6.

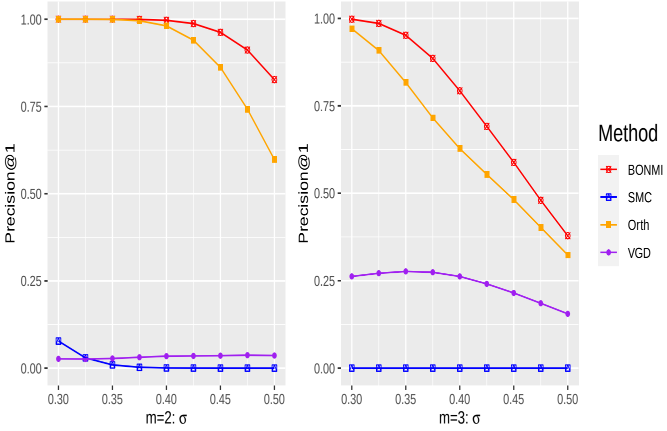

Figure 4: Simulation results of settings (i) and (ii). The relative estimation errors of $\mathbf{W}_0^*$ are presented. setting (iii): fix $p_0 = 0.1$ and range σ from 0.3 to 0.5.
